# Incontinentia pigmenti underlies thymic dysplasia, autoantibodies to type I IFNs, and viral diseases

**DOI:** 10.1084/jem.20231152

**Published:** 2024-10-01

**Authors:** Jérémie Rosain, Tom Le Voyer, Xian Liu, Adrian Gervais, Laura Polivka, Axel Cederholm, Laureline Berteloot, Audrey V. Parent, Alessandra Pescatore, Ezia Spinosa, Snezana Minic, Ana Elisa Kiszewski, Miyuki Tsumura, Chloé Thibault, Maria Esnaola Azcoiti, Jelena Martinovic, Quentin Philippot, Taushif Khan, Astrid Marchal, Bénédicte Charmeteau-De Muylder, Lucy Bizien, Caroline Deswarte, Lillia Hadjem, Marie-Odile Fauvarque, Karim Dorgham, Daniel Eriksson, Emilia Liana Falcone, Mathilde Puel, Sinem Ünal, Amyrath Geraldo, Corentin Le Floc’h, Hailun Li, Sylvie Rheault, Christine Muti, Claire Bobrie-Moyrand, Anne Welfringer-Morin, Ramsay L. Fuleihan, Romain Lévy, Marie Roelens, Liwei Gao, Marie Materna, Silvia Pellegrini, Lorenzo Piemonti, Emilie Catherinot, Jean-Christophe Goffard, Arnaud Fekkar, Aissata Sacko-Sow, Camille Soudée, Soraya Boucherit, Anna-Lena Neehus, Cristina Has, Stefanie Hübner, Géraldine Blanchard-Rohner, Blanca Amador-Borrero, Takanori Utsumi, Maki Taniguchi, Hiroo Tani, Kazushi Izawa, Takahiro Yasumi, Sotaro Kanai, Mélanie Migaud, Mélodie Aubart, Nathalie Lambert, Guy Gorochov, Capucine Picard, Claire Soudais, Anne-Sophie L’Honneur, Flore Rozenberg, Joshua D. Milner, Shen-Ying Zhang, Pierre Vabres, Dusan Trpinac, Nico Marr, Nathalie Boddaert, Isabelle Desguerre, Manolis Pasparakis, Corey N. Miller, Cláudia S. Poziomczyk, Laurent Abel, Satoshi Okada, Emmanuelle Jouanguy, Rémi Cheynier, Qian Zhang, Aurélie Cobat, Vivien Béziat, Bertrand Boisson, Julie Steffann, Francesca Fusco, Matilde Valeria Ursini, Smail Hadj-Rabia, Christine Bodemer, Jacinta Bustamante, Hervé Luche, Anne Puel, Gilles Courtois, Paul Bastard, Nils Landegren, Mark S. Anderson, Jean-Laurent Casanova

**Affiliations:** 1 Laboratory of Human Genetics of Infectious Diseases, Necker Branch, Inserm U1163, Necker Hospital for Sick Children, Paris, France; 2 Imagine Institute, University of Paris Cité, Paris, France; 3 St. Giles Laboratory of Human Genetics of Infectious Diseases, Rockefeller Branch, The Rockefeller University, New York, NY, USA; 4 Study Center for Primary Immunodeficiencies, Necker Hospital for Sick Children, Assistance Publique-Hôpitaux de Paris (AP-HP), Paris, France; 5 Clinical Immunology Department, AP-HP, Saint-Louis Hospital, Paris, France; 6 Diabetes Center, University of California San Francisco, San Francisco, CA, USA; 7 Department of Dermatology, Reference Center for Genodermatosis and Rare Skin Diseases (MAGEC), University of Paris Cité, Necker Hospital for Sick Children, AP-HP, Paris, France; 8 Reference Center for Mastocytosis (CEREMAST), Necker Hospital for Sick Children, AP-HP, Paris, France; 9 Science for Life Laboratory, Department of Medical Biochemistry and Microbiology, Uppsala University, Uppsala, Sweden; 10 Pediatric Radiology Department, Necker Hospital for Sick Children, Imagine Inserm Institute, U1163, AP-HP, Paris, France; 11 Institute of Genetics and Biophysics “Adriano Buzzati-Traverso,” IGB-CNR, Naples, Italy; 12 Clinics of Dermatovenerology, Clinical Center of Serbia, Belgrade, Serbia; 13 School of Medicine, University of Belgrade, Belgrade, Serbia; 14 Section of Dermatology, Federal University of Health Sciences of Porto Alegre, Porto Alegre, Brazil; 15 Section of Pediatric Dermatology, Hospital da Criança Santo Antônio, Irmandade da Santa Casa de Misericórdia de Porto Alegre, Porto Alegre, Brazil; 16 Hiroshima University Graduate School of Biomedical and Health Sciences, Hiroshima, Japan; 17 Unit of Fetal Pathology, Hospital Antoine Béclère, Paris Saclay University, Paris, France; 18 Department of Immunology, Sidra Medicine, Doha, Qatar; 19 University of Paris Cité, CNRS, Inserm, Institut Cochin, Paris, France; 20 Immunophenomics Center (CIPHE), Aix Marseille University, Inserm, CNRS, Marseille, France; 21 University Grenoble Alpes, CEA, Inserm, BGE UA13, Grenoble, France; 22 Sorbonne University, Inserm, Centre for Immunology and Microbial Infections, CIMI-Paris, Paris, France; 23 Department of Immunology, Genetics and Pathology, Uppsala University, Uppsala, Sweden; 24 Center for Immunity, Inflammation and Infectious Diseases, Montréal Clinical Research Institute (IRCM), Montréal, Canada; 25 Department of Medicine, Montréal University, Montréal, Canada; 26 Center of Research of the Geriatric University Institute of Montréal, University of Montréal, Montréal, Canada; 27 Department of Genetics, André Mignot Hospital, Versailles, France; 28 Department of Obstetrics and Gynecology, André Mignot Hospital, Versailles, France; 29 Department of Pediatrics, Columbia University Medical Center, New York, NY, USA; 30 Pediatric Hematology-Immunology and Rheumatology Unit, Necker Hospital for Sick Children, AP-HP, Paris, France; 31 Diabetes Research Institute, IRCCS Ospedale San Raffaele, Milan, Italy; 32 Department of Respiratory Diseases, Foch Hospital, Suresnes, France; 33 Internal Medicine, Brussels University Hospital, Free University of Brussels, Anderlecht, Belgium; 34 Department of Parasitology Mycology, Pitié-Salpêtrière Hospital, AP-HP, Paris, France; 35 Department of Pediatrics, Jean Verdier Hospital, AP-HP, Bondy, France; 36 Department of Dermatology, Medical Center-University of Freiburg, Freiburg im Breisgau, Germany; 37 European Reference Network (ERN) for Rare and Undiagnosed Skin Disorders; 38 Unit of Immunology, Vaccinology, and Rheumatology, Division of General Pediatrics, Department of Woman, Child, and Adolescent Medicine, Geneva University Hospitals and Faculty of Medicine, University of Geneva, Geneva, Switzerland; 39 Internal Medicine Department, Lariboisière Hospital, AP-HP, University of Paris Cité, Paris, France; 40 Department of Pediatrics, Hiroshima University Hospital, Hiroshima, Japan; 41 Department of Pediatrics, Hiroshima Prefectural Hospital, Hiroshima, Japan; 42 Department of Pediatrics, Kyoto University Graduate School of Medicine, Kyoto, Japan; 43 Division of Child Neurology, Department of Brain and Neurosciences, Faculty of Medicine, Tottori University, Yonago, Japan; 44 Departments of Pediatric Neurology, Necker Hospital for Sick Children, AP-HP, University of Paris Cité, Paris, France; 45 Department of Immunology, Pitié-Salpêtrière Hospital, AP-HP, Paris, France; 46 Laboratory of Lymphocyte Activation and Susceptibility to EBV Infection, Inserm U1163, Paris, France; 47 Department of Virology, University of Paris Cité and Cochin Hospital, AP-HP, Paris, France; 48 MAGEC Reference Center for Rare Skin Diseases, Dijon Bourgogne University Hospital, Dijon, France; 49 Institute of Histology and Embryology, School of Medicine, University of Belgrade, Belgrade, Serbia; 50 College of Health and Life Sciences, Hamad Bin Khalifa University, Doha, Qatar; 51 Institute for Genetics, University of Cologne, Cologne, Germany; 52 Private Dermatology Practice, Porto Alegre, Brazil; 53 Department of Genomic Medicine, Necker Hospital for Sick Children, AP-HP, University of Paris Cité, Paris, France; 54 Center for Molecular Medicine, Department of Medicine (Solna), Karolinska Institute, Stockholm, Sweden; 55 Department of Pediatrics, Necker Hospital for Sick Children, AP-HP, Paris, France; 56 Howard Hughes Medical Institute, New York, NY, USA

## Abstract

Human inborn errors of thymic T cell tolerance underlie the production of autoantibodies (auto-Abs) neutralizing type I IFNs, which predispose to severe viral diseases. We analyze 131 female patients with X-linked dominant incontinentia pigmenti (IP), heterozygous for loss-of-function (LOF) *NEMO* variants, from 99 kindreds in 10 countries. Forty-seven of these patients (36%) have auto-Abs neutralizing IFN-α and/or IFN-ω, a proportion 23 times higher than that for age-matched female controls. This proportion remains stable from the age of 6 years onward. On imaging, female patients with IP have a small, abnormally structured thymus. Auto-Abs against type I IFNs confer a predisposition to life-threatening viral diseases. By contrast, patients with IP lacking auto-Abs against type I IFNs are at no particular risk of viral disease. These results suggest that IP accelerates thymic involution, thereby underlying the production of auto-Abs neutralizing type I IFNs in at least a third of female patients with IP, predisposing them to life-threatening viral diseases.

## Teaser

Heterozygosity for null *NEMO* mutations underlies hypotrophic and dysplastic thymus in mice and humans. Women with incontinentia pigmenti therefore have a high prevalence of autoantibodies neutralizing type I interferons, which predisposes them to severe viral diseases.

## Introduction

Autoantibodies (auto-Abs) neutralizing type I interferons (IFNs) were first reported in 1981 in a patient treated with recombinant type I IFN (Vallbracht et al., 1981). They were soon reported in other patients treated with type I IFN (Ikeda et al., 1989; Protzman et al., 1984; Bonetti et al., 1994), and in patients with myasthenia gravis (Bello-Rivero et al., 2004), thymoma (Burbelo et al., 2010), and systemic lupus erythematosus (Gupta et al., 2016; Howe and Leung, 2019; Panem et al., 1982). Despite their detection in a single patient with disseminated zoster in 1981 (Mogensen et al., 1981) and 1984 (Pozzetto et al., 1984), these auto-Abs were long thought to be clinically silent in terms of susceptibility to viral diseases (Meyer et al., 2016). However, this perception changed in 2020, when auto-Abs against type I IFNs were found to underlie about 20% of COVID-19 pneumonia fatal cases (Bastard et al., 2020, 2021a; Zhang et al., 2022a; Manry et al., 2022), 25% of hospitalizations for Middle East respiratory syndrome (MERS) (Alotaibi et al., 2023), about 5% of cases of life-threatening influenza pneumonia (Zhang et al., 2022b), 35% of cases of life-threatening adverse reaction to yellow fever live-attenuated vaccine (YFV) (Bastard et al., 2021d), and 40% of cases of West Nile virus (WNV) encephalitis (Gervais et al., 2023). They were also found to increase the risk of cutaneous infections with HSV-1 and VZV in patients with various conditions (Walter et al., 2015; Hetemäki et al., 2021b; Busnadiego et al., 2022). These results were replicated by various approaches in >30 cohorts worldwide for COVID-19 (Busnadiego et al., 2022; Abers et al., 2021; Acosta-Ampudia et al., 2021; Bastard et al., 2021e, 2024b; Chang et al., 2021; Chauvineau-Grenier et al., 2022; Goncalves et al., 2021; Koning et al., 2021; Solanich et al., 2021; Troya et al., 2021; Vazquez et al., 2021; van der Wijst et al., 2021; Schidlowski et al., 2022; Wang et al., 2021; Mathian et al., 2022; Lemarquis et al., 2021; Meisel et al., 2021; Savvateeva et al., 2021; Ziegler et al., 2021; Carapito et al., 2022; Credle et al., 2022; Eto et al., 2022; Frasca et al., 2022; Lamacchia et al., 2022; Raadsen et al., 2022; Simula et al., 2022; Soltani-Zangbar et al., 2022; Akbil et al., 2022; Borsani et al., 2022; Imberti et al., 2023; Philippot et al., 2023; Saheb Sharif-Askari et al., 2023; Pons et al., 2023) and in a single cohort for YFV (Le Hir et al., 2024).

Plasma containing such auto-Abs (diluted 1/10) can neutralize low (100 pg/ml) or high (10 ng/ml) concentrations of the 12 types of IFN-α (encoded by 13 loci) and/or the single IFN-ω, and more rarely IFN-β (Bastard et al., 2020, 2021a, 2021d, 2021e). The production of these auto-Abs can be genetically driven in patients with rare inborn errors of immunity (IEI), as best exemplified by their occurrence in most, if not all patients with auto-immune polyendocrinopathy syndrome type 1 (APS-1) (Meyer et al., 2016; Hetemäki et al., 2021b; Bastard et al., 2021e; Meager et al., 2006; Oftedal et al., 2015; Valenzise et al., 2023), which is autosomal recessive (AR) or dominant (AD) and due to rare loss-of-function (LOF) variants of *AIRE.* These auto-Abs have also been found in some patients with AR partial RAG1 or RAG2 deficiency (Walter et al., 2015), in one-third of patients with X-linked recessive FOXP3 deficiency (Rosenberg et al., 2018), and in almost all patients with inborn errors of the alternative NF-κB pathway (Maccari et al., 2017; Ramakrishnan et al., 2018; Parsons et al., 2019; Le Voyer et al., 2023). All these disorders impair thymopoiesis and T cell tolerance due to their expression in thymocytes or medullary thymic epithelial cells (mTECs). In addition, one patient with IKZF2 (Hetemäki et al., 2021a) and one with pre-TCR-α deficiency (Materna et al., 2024) have been reported to have auto-Abs against type I IFNs. Both these proteins are normally expressed by thymocytes. Environmental factors contributing to the development of auto-Abs against type I IFNs are less well known; previous viral infections may be required through their induction of type I IFNs (Fernbach et al., 2024; Wells et al., 2024; Hale, 2023).

The first patients with auto-Abs against type I IFNs and life-threatening COVID-19 to be described (Bastard et al., 2020) included a woman with incontinentia pigmenti (IP). Moreover, some uninfected French IP patients tested by ELISA were found to have auto-Abs against type I IFNs (Bastard et al., 2020). This result came as a surprise because IP had not previously been associated with autoimmune or viral diseases (Fusco et al., 2014; Hübner et al., 2022; Bodemer et al., 2020). IP (OMIM: 308300) is an X-linked dominant ectodermal dysplasia caused by LOF germline variants of the *IKBKG/NEMO* gene (Smahi et al., 2000; Conte et al., 2014; Fusco et al., 2008). In female patients heterozygous for such variants, IP is characterized by abnormalities in ectoderm-derived tissues, including the skin, eyes, teeth, hair, breasts, nails, and central nervous system (CNS) (Bodemer et al., 2020; Scheuerle and Ursini, 1993). Skin lesions progress through four successive stages, culminating in atrophy/hypopigmentation (Bodemer et al., 2020; Scheuerle and Ursini, 1993). Severe disease is associated with the occurrence of vascular lesions with ophthalmological and neurological consequences, mostly occurring in early infancy (Bodemer et al., 2020; Scheuerle and Ursini, 1993). All patients with IP have skin lesions, whereas only about 30% also have neurological manifestations and 30% have ophthalmologic manifestations (Bodemer et al., 2020; Scheuerle and Ursini, 1993; Minić et al., 2014). In the absence of ophthalmological and neurosensorial vasculopathy sequelae, adult women with IP are healthy (Scheuerle, 2019) and some therefore remain undiagnosed (Hadj-Rabia et al., 2011).

The prevalence of IP in the general population is estimated at about 1/100,000 (Scheuerle and Ursini, 1993; Bodemer et al., 2020; Minić et al., 2022). IP is often sporadic, with 65% of causal variants arising de novo (Smahi et al., 2000; Conte et al., 2014; Fusco et al., 2008). More than 75% of IP cases are due to a recurrent 11.7 kb deletion encompassing exons 4–10 (Δ4–10) of *NEMO* (Smahi et al., 2000; Conte et al., 2014; Fusco et al., 2008). The other pathogenic variants are more diverse (Smahi et al., 2000; Conte et al., 2014; Fusco et al., 2008). The genetic basis of IP is unknown in a minority of cases possibly due to difficulties analyzing the *NEMO* locus due to a nearby homologous pseudogene (Smahi et al., 2000; Conte et al., 2014). Female patients with IP usually display completely skewed X-linked chromosome inactivation in various cells, particularly fibroblasts and peripheral blood cells (Parrish et al., 1996). The mechanism of disease in the skin and vessels is thought to involve TNF-dependent sensitivity to the apoptosis of cells expressing the mutant *NEMO* allele (Senegas et al., 2015; Pescatore et al., 2016; Pasparakis et al., 2002; Nenci et al., 2006). Consistently, women with IP are often healthy, and even fertile, whereas IP is lethal in hemizygous male fetuses in utero, presumably due to massive apoptosis (Senegas et al., 2015). However, some boys with IP have been diagnosed, most having a 47,XXY karyotype or somatic mosaicism (Kenwrick et al., 2001). We further explored the potential link between a type I IFNs defect and overall health in female patients with IP by investigating the prevalence, consequences, and pathogenic mechanism of auto-Abs against type I IFNs in a large international cohort of IP patients.

## Results

### Baseline characteristics of an international cohort of female patients with IP

**Figure S1.**
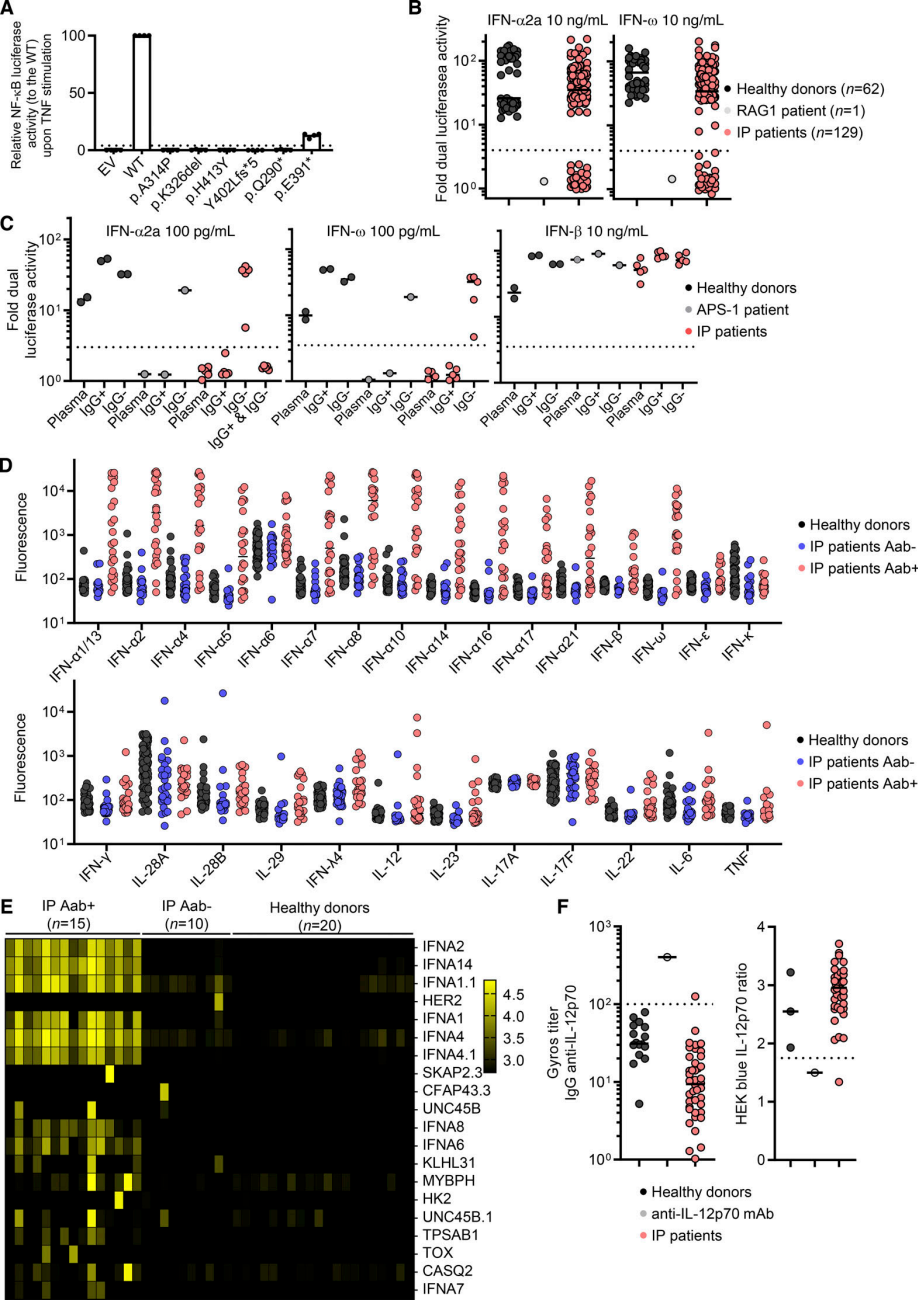
**Auto-antibodies to type I IFNs and other cytokines in females with incontinentia pigmenti.** Related to Fig. 1. **(A)** Dual luciferase NF-κB activity of HEK293T cells transfected with empty vector (EV) or a vector containing WT NEMO cDNA, or various NEMO variants. Cells were stimulated with TNF. Results are normalized against the WT. The p.A314P, p.K326del, p.H413Y and p.Y402Lfs*5 variants were found in IP patients from this cohort. p.Q290* was previously described in a patient with IP and was used as an amorphic control. p.E391* was previously described in a patient with ectodermal dysplasia and immunodeficiency and was used as a hypomorphic control. Pooled results for from *n* = 4 independent experiments are presented. Bar is displayed at 4%. **(B)** Neutralization of 10 ng/ml IFN-α2a or IFN-ω by a 1:10 dilution of plasma from the indicated individuals or patients. All patients with neutralizing ratio below 3 were assessed two or three times independently. **(C)** Neutralization of 100 pg/ml IFN-α2a, 100 pg/ml IFN-ω, or 10 ng/ml of IFN-β by a 1:10 dilution of plasma, the purified IgG-positive fraction (IgG^+^), or the purified IgG-depleted fraction (IgG^−^) from the indicated individuals or patients. **(D)** Bead-based protein array detecting IgG auto-Abs against the indicated cytokines in *n* = 93 healthy donors, and in *n* = 30 and *n* = 24 IP patients negative and positive, respectively, for the neutralization of type I IFNs by auto-Abs. **(E)** Anti-human IgG fluorescence signal intensities (log_10_) according to protein microarray (HuProt) data for all individuals plotted with correlation clusters for proteins, including the 20 proteins with the largest positive fold-change in levels. Case-control status is indicated at the top. Data displayed are merged from two independent experiments. **(F)** Gyros anti-IL-12p70 IgG antibody titer (left) and neutralization activity determined in HEK293-blue cells (right) for plasma from healthy donors and patients with IP.

We enrolled 131 female patients with clinically proven IP from 99 kindreds originating and living in 10 different countries: Belgium (7 patients from 3 kindreds), Brazil (7 patients from 6 kindreds), Canada (1 patient from 1 kindred), France (65 patients from 51 kindreds), Germany (2 patients from 2 kindreds), Italy (22 patients from 19 kindreds), Japan (7 patients from 6 kindreds), Serbia (13 patients from 6 kindreds), Switzerland (1 patient from 1 kindred), and the United States of America (6 patients from 4 kindreds) (Table S1). Patient age ranged from 3 wk to 84 years, with a mean age of 20 years. IP was sporadic in 55% of patients (47 out of 85) and familial in 45% (38/85). Cutaneous manifestations were observed in all patients (131 of 131 [100%]), ophthalmological complications were observed in 34% (35/103), and neurological impairment was observed in 30% (32/105). A recurrent deletion (Δ4–10) in *IKBKG*/*NEMO* (NM_001099857) was found in 108 patients from 79 kindreds, corresponding to 82% of patients and 80% of kindreds, respectively. Ten other variants were found in 14 patients from 11 kindreds: *n* = 7 frameshift indels or nonsense variants (c.628_651delinsCG, c.646del, p.Y241*, c.756_765del, c.850del, c.1110dup, and c.1204dup) each found in one or two patients from a single kindred, *n* = 1 in-frame deletion (p.K326del) in three patients, and *n* = 2 missense variants (p.A314P and p.H413Y). Each of these other variants was found in one kindred, except for the p.K326del variant, which was found in two unrelated kindreds. None of these 10 variants were previously experimentally tested. Six of these variants are frameshift or nonsense variants (c.628_651delinsCG, c.646del, p.Y241*, c.756_765del, c.850del, and c.1110dup) predicted to be LOF (pLOF) as they lead to truncations within the first 350 amino acids, i.e., at positions at which truncations are known to be lethal in hemizygous males and associated with IP in heterozygous females (Smahi et al., 2000; Fusco et al., 2008, 2012; Conte et al., 2014). We used a TNF-dependent NF-κB reporter assay to test the other four variants: the in-frame deletion (p.K326del), the two missense variants (p.A314P and p.H413Y), and a pLOF but distal truncating variant c.1204dup (p.Y402Lfs*5). All of these variants were found to be LOF (Fig. S1 A). The causal variant was unknown in *n* = 9 patients from nine kindreds. We established an international cohort of IP patients with the canonical clinical and genetic features of IP (Fusco et al., 2014; Hübner et al., 2022).

### High prevalence of auto-Abs neutralizing type I IFNs in female patients with IP

**Figure 1.**
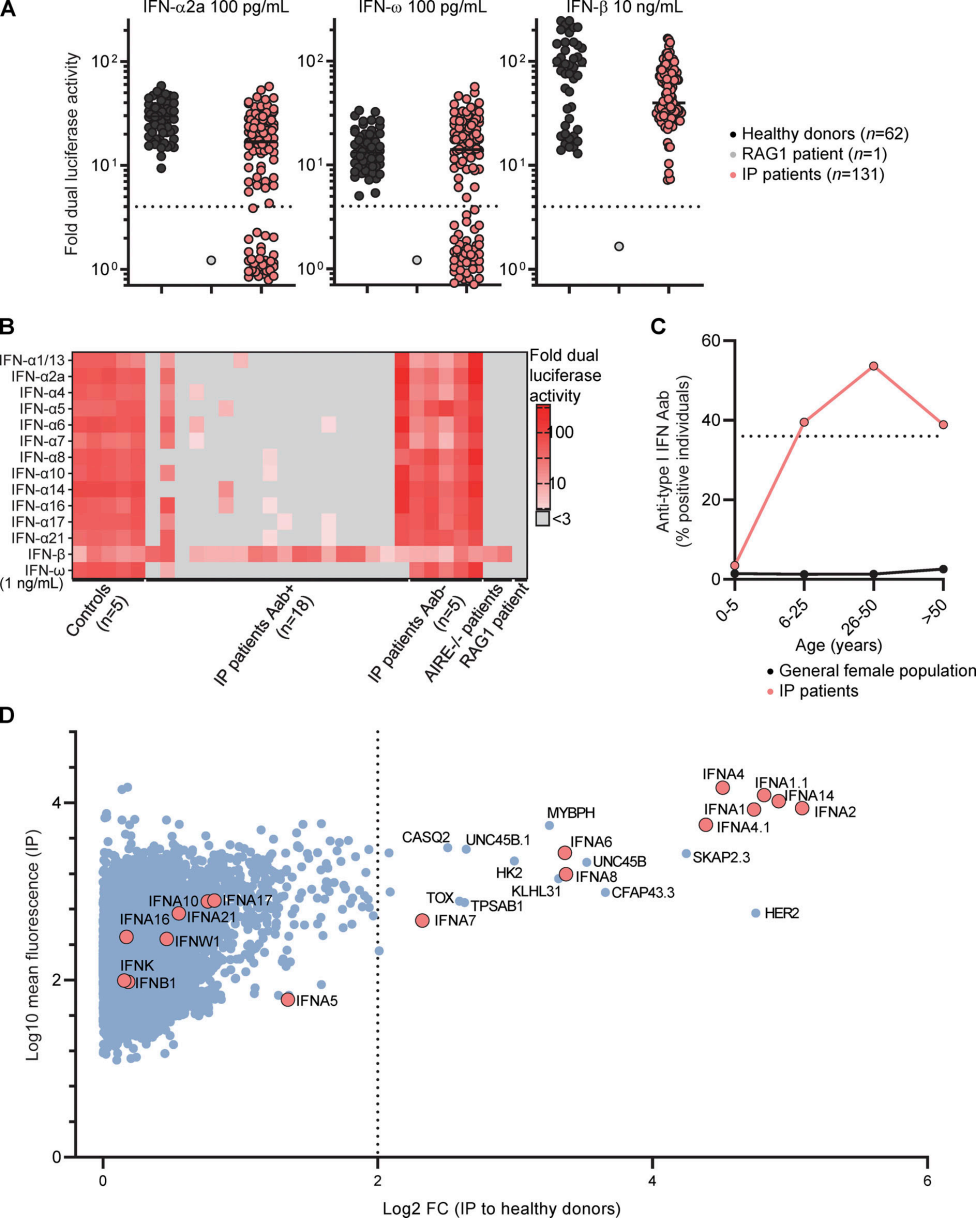
**Auto-Abs against type I IFNs in female patients with IP. (A)** Neutralization of 100 pg/ml IFN-α2a, 100 pg/ml IFN-ω, and 10 ng/ml IFN-β by a 1:10 dilution of plasma from the indicated individuals or patients. All patients with a neutralizing ratio below 3 were assessed two or three times independently. **(B)** Neutralization of 1 ng/ml of 15 type I IFNs by plasma diluted 1:10. **(C)** The proportion of individuals with plasma-neutralizing type I IFNs by age, for female patients with IP and the general female population. **(D)** Anti-human IgG fluorescence signal intensities with positive fold-change in 15 IP patients with serum neutralizing type I IFNs and 10 IP patients without auto-Abs neutralizing type I IFNs, relative to 20 healthy donors. Each dot corresponds to the protein spot with the highest signal intensity among the duplicate spots for the protein concerned. Data displayed are merged from two independent experiments.

We assessed the prevalence of auto-Abs in IP patients by testing plasma samples from all the patients of the cohort for neutralizing activity in a luciferase IFN-stimulated response element (ISRE) reporter assay. Plasma samples were diluted 1:10 and incubated in the presence of type I IFNs (IFN-α2a or IFN-ω) at two different concentrations (10 ng/ml and 100 pg/ml). We also screened for neutralizing activity against IFN-β at a concentration of 10 ng/ml. We observed neutralizing activity against 10 ng/ml IFN-α2a in 21% of patients (27/131), 100 pg/ml IFN-α2a in 25% of patients (33/131), 10 ng/ml IFN-ω in 21% of patients (28/131), 100 pg/ml IFN-ω in 34% of patients (44/131), and 10 ng/ml IFN-β in 0% of patients (0/131) (Fig. 1 A and Fig. S1 B). Overall, 36% of the IP patients tested (47/131) had auto-Abs neutralizing IFN-α2a and/or IFN-ω, 26% (34/131) had auto-Abs neutralizing both, <1% (1/131) had auto-Abs neutralizing only IFN-α2a, and 10% (13/131) had auto-Abs neutralizing only IFN-ω. IgG purification for five patients showed that the neutralization of IFN-α2a and IFN-ω was mediated by the IgG fraction of plasma rather than by plasma from which IgG was deleted, confirming bona fide neutralization by IgG auto-Abs (Fig. S1 C). In addition, for patients positive and negative for type I IFNs neutralization, we obtained positive and negative results, respectively, in bead-based protein array screening for IgG directed against IFN-α subtypes and/or IFN-ω (Fig. S1 D). Finally, we tested for neutralizing activity against 12 different IFN-α subtypes in 18 patients with auto-Abs neutralizing IFN-α2b or IFN-ω. Plasma from all these patients neutralized some or all the other subtypes of IFN-α at a concentration of 1 pg/ml (Fig. 1 B). IP patients, thus, had a strikingly high prevalence of auto-Abs against type I IFNs neutralizing IFN-α subtypes and/or IFN-ω, and this prevalence (∼40%) was stable from the age of 6 years onwards (Fig. 1 C). The odds ratios for carrying such auto-Abs neutralizing high (10 ng/ml) or low concentrations (100 pg/ml) in IP female patients relative to the age-adjusted general female population were 126 (95% confidence interval [CI]: 75–212) and 45 (95% CI: 29–72), respectively.

### Absence of broad autoimmune reactivity in female patients with IP

Given the high prevalence of auto-Abs against type I IFNs in female patients with IP, we investigated the possibility of broader autoimmunity in these individuals. To this end, we performed a proteome-wide auto-Abs repertoire analysis, assessing IgG reactivity to a panel of 20,052 native full-length human proteins expressed in yeast. In this analysis, we included 15 IP patients who had tested positive and 10 IP patients who had tested negative for neutralizing auto-Abs against type I IFNs. In IP patients positive for neutralizing auto-Abs against type I IFNs, autoreactivity was clearly strongest against various type I IFNs, including IFN-α1, IFN-α2, IFN-α4, IFN-α6, IFN-α7, IFN-α8, and IFN-α14 (Fig. 1 D, Fig. S1 E, and Table S2). Positive reactivity to these targets was observed specifically in the IP patients whose serum samples neutralized type I IFNs. A few other hits common to multiple IP patients were detected, including KLHL31 and UNC45B, detected in four and three patients, respectively. However, these hits were not confirmed in the validation experiment performed with bead-based protein arrays, suggesting they were probably false positives (Table S3). In addition, one patient tested positive for anti-TNF antibodies and another for anti-HER2 antibodies in protein microarray screening; in both cases, these positive results were explained by therapeutic use of the corresponding monoclonal antibodies (Fig. S1 E and Table S2). One patient tested positive for autoimmune reactivity to IL-12p70 antigen, which was confirmed by measurements of anti-IL12p70 IgG levels and a specific neutralizing assay (Fig. S1 F). Positivity for auto-Abs against this cytokine appeared to be isolated, as the screening of plasma from another 40 IP patients revealed no additional cases of strong IgG reactivity to IL-12p70 in terms of Gyros titer or neutralizing IL-12p70 activity (Fig. S1 F). No strong enrichment in auto-Abs was found for any cytokine other than type I IFNs and IL-12p70 in proteome microarrays (Fig. 1 D, Fig. S1 E, and Table S2) and bead-based protein arrays (Fig. S1 D and Table S3). Finally, 10 IP patients positive for auto-Abs against type I IFNs tested negative for auto-Abs against native DNA, extractable nuclear antigens (RNP, Sm, SSa, SSB, Scl70, Jo1), and tissue antigens (smooth muscle, mitochondria and LKM1). Thus, the auto-Ab repertoires of IP patients were almost entirely restricted to auto-Abs against type I IFNs.

### Natural viral infections and reactions to live-attenuated viral vaccines

**Figure 2.**
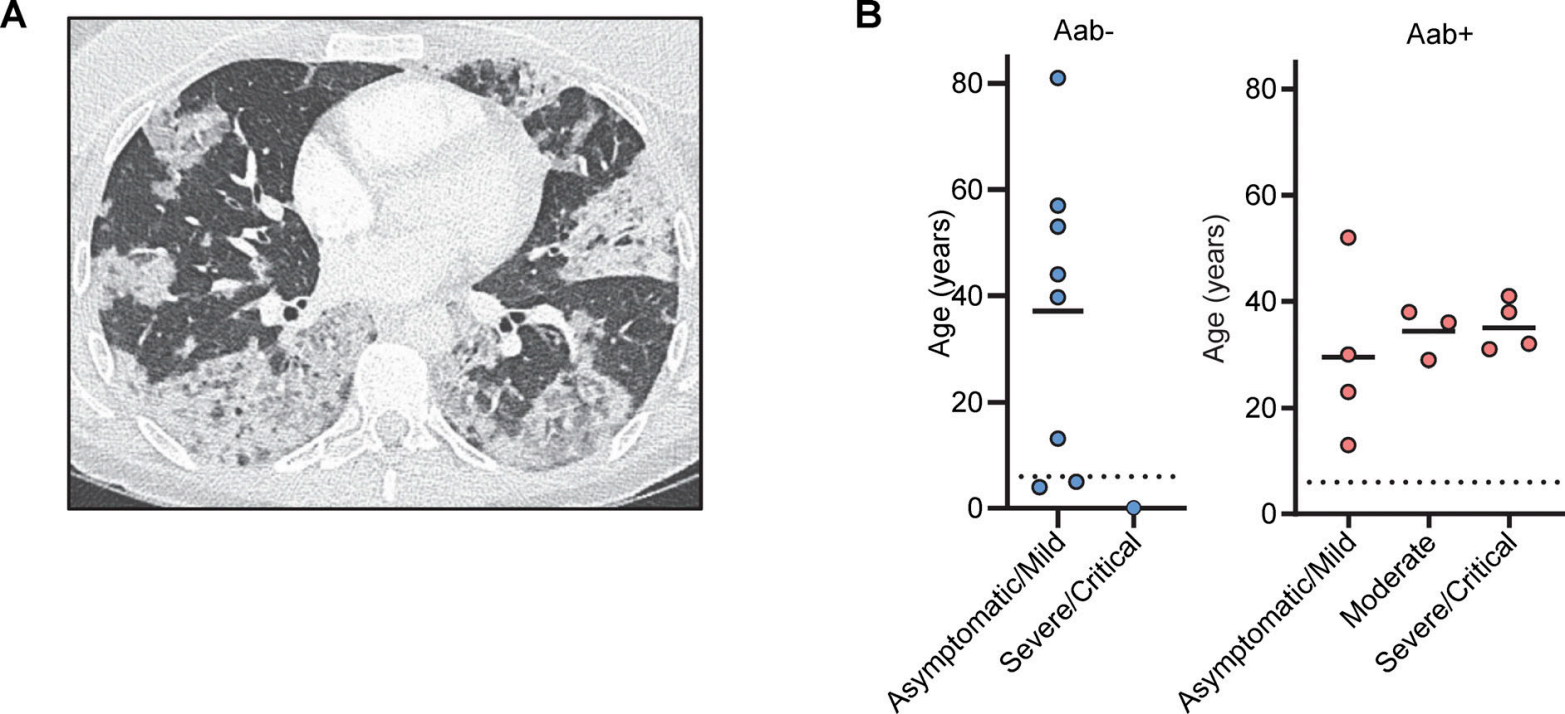
**Viral diseases in female patients with IP. (A)** Thorax-computed tomography scan for P1 who had critical COVID-19 pneumonia. **(B)** Severity of COVID-19 before vaccination as a function of negativity or positivity for auto-Abs against type I IFNs (Aab−, and Aab+, respectively), and age. One of the patients (P9) was excluded from this figure because she received early treatment with recombinant IFN-β (Bastard et al., 2021b).

**Figure S2.**
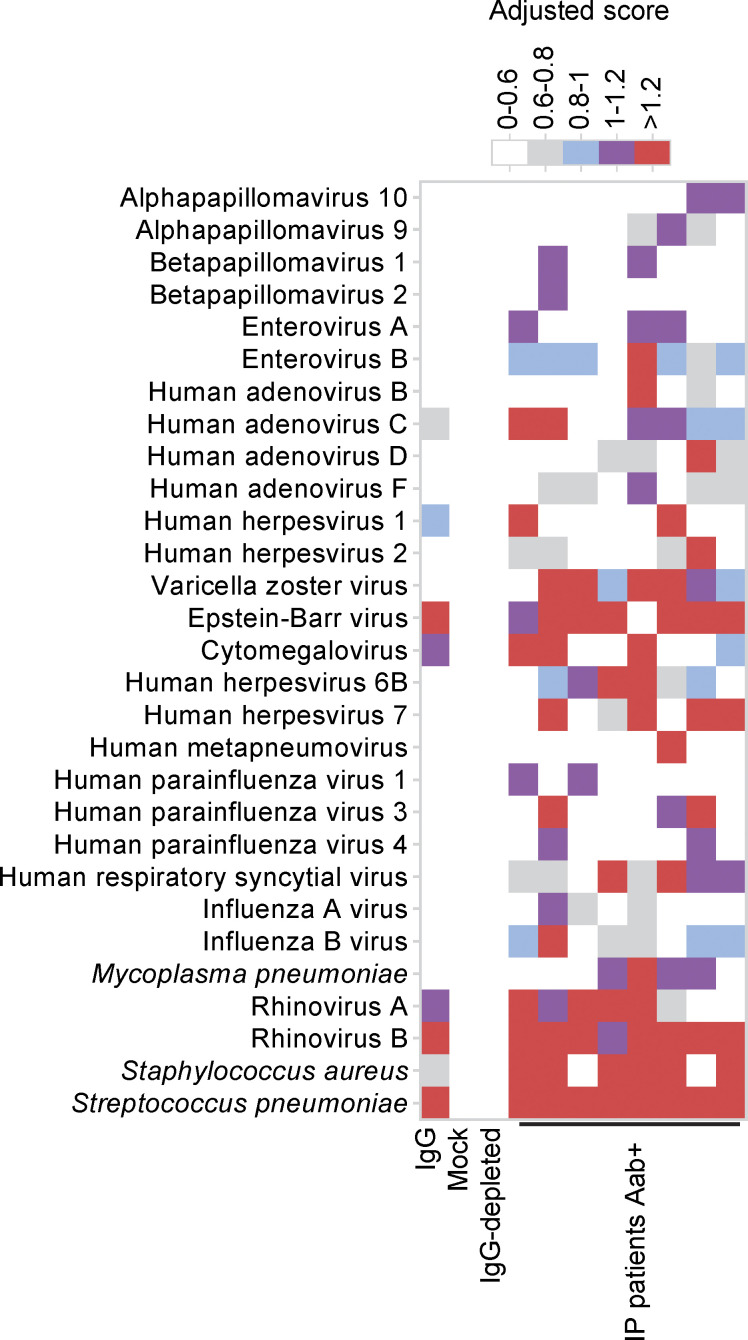
**Exposure to viruses and bacteria in females with incontinentia pigmenti.** Related to Fig. 2. Antiviral antibody responses to species for which at least one sample tested seropositive by PhIP-Seq, based on in-house stringent cutoff values, color-coded as indicated.

The presence of auto-Abs neutralizing type I IFNs confers a predisposition to several viral diseases: COVID-19 pneumonia (Bastard et al., 2020, 2021a, 2021e; Zhang et al., 2022a; Busnadiego et al., 2022; Abers et al., 2021; Acosta-Ampudia et al., 2021; Chang et al., 2021; Chauvineau-Grenier et al., 2022; Goncalves et al., 2021; Koning et al., 2021; Solanich et al., 2021; Troya et al., 2021; Vazquez et al., 2021; van der Wijst et al., 2021; Schidlowski et al., 2022; Wang et al., 2021; Mathian et al., 2022; Lemarquis et al., 2021; Meisel et al., 2021; Savvateeva et al., 2021; Ziegler et al., 2021; Carapito et al., 2022; Credle et al., 2022; Eto et al., 2022; Frasca et al., 2022; Lamacchia et al., 2022; Raadsen et al., 2022; Simula et al., 2022; Soltani-Zangbar et al., 2022; Akbil et al., 2022; Borsani et al., 2022), MERS pneumonia (Alotaibi et al., 2023), influenza pneumonia (Zhang et al., 2022b), WNV encephalitis (Gervais et al., 2023), cutaneous infections due to HSV-1 and VZV (Hetemäki et al., 2021b; Busnadiego et al., 2022), and severe adverse reaction to live-attenuated YFV vaccine (Bastard et al., 2021d). We therefore recorded the history of viral disease and the outcome of administrations of live-attenuated vaccines for our IP cohort. Eleven patients with auto-Abs were exposed to SARS-CoV-2 before vaccination (see Materials and methods for detailed case reports). Four of these 11 patients had critical or severe forms requiring for oxygen support (P1 [Bastard et al., 2020], P2 [Rheault, 2021], P3, and P4) (Fig. 2, A and B); three patients had a moderate form with pneumonia but no requirement for oxygen support (P5, P6, and P11), and four patients presented mild or asymptomatic forms (P7, P8, P9, and P10). One patient with a mild form (P9) was treated with recombinant IFN-β early in the infection (Bastard et al., 2021b). Overall, two-third of IP patients with auto-Abs had COVID-19 pneumonia before vaccination, a proportion similar to that reported in other cohorts of patients with auto-Abs against type I IFNs, such as patients with APS-1 (Bastard et al., 2021e; Schidlowski et al., 2022; Lemarquis et al., 2021; Meisel et al., 2021; Carpino et al., 2021; Beccuti et al., 2020; Ferré et al., 2021; Valenzise et al., 2023), or inborn errors of the alternative NF-κB pathway (Le Voyer et al., 2023). One patient with auto-Abs against type I IFNs (P3) had a history of H_1_N_1_ influenza viral pneumonia requiring hospitalization but not oxygen support. No other relevant history of severe non-SARS-CoV-2 viral disease was noted among the patients positive for auto-Abs, despite their exposure to multiple RNA and DNA viruses, including respiratory viruses and Herpesviridae*,* such as VZV, as demonstrated by phage immunoprecipitation sequencing (PhIP-Seq) in seven patients (Fig. S2). One patient (P12) had a history of YFV-associated neurological disease following vaccination with live-attenuated YFV at the age of 54 years (see Materials and methods for detailed case reports). Four other patients with auto-Abs had been vaccinated against YFV at the ages of 10, 20, 27, and 31 years, and none of these patients required hospitalization after vaccination. However, one of these patients, aged 20 years at the time of vaccination, presented fever and asthenia for 1 wk after the administration of YFV. There was no history of adverse reaction to MMR vaccination in the 20 auto-Ab–positive patients who received this vaccine. Finally, there was no history of life-threatening viral infection or adverse reaction to live viral vaccine in IP patients negative for auto-Abs, nine of whom had positive serological tests for SARS-CoV-2 before vaccination, except for critical COVID-19 pneumonia in a 2-wk-old patient (Fig. 2 B). This patient received steroid treatment for IP during the first few days of life. Given her age, this patient would, in any case, have been unlikely to have already produced auto-Abs that could account for the development of COVID-19. Thus, a history of life-threatening forms of COVID-19, influenza pneumonia, and severe adverse reaction to YFV was found in the IP patients positive for auto-Abs against type I IFNs, but not in those without such antibodies.

### Determinants of the presence of auto-antibodies in IP patients

**Figure 3.**
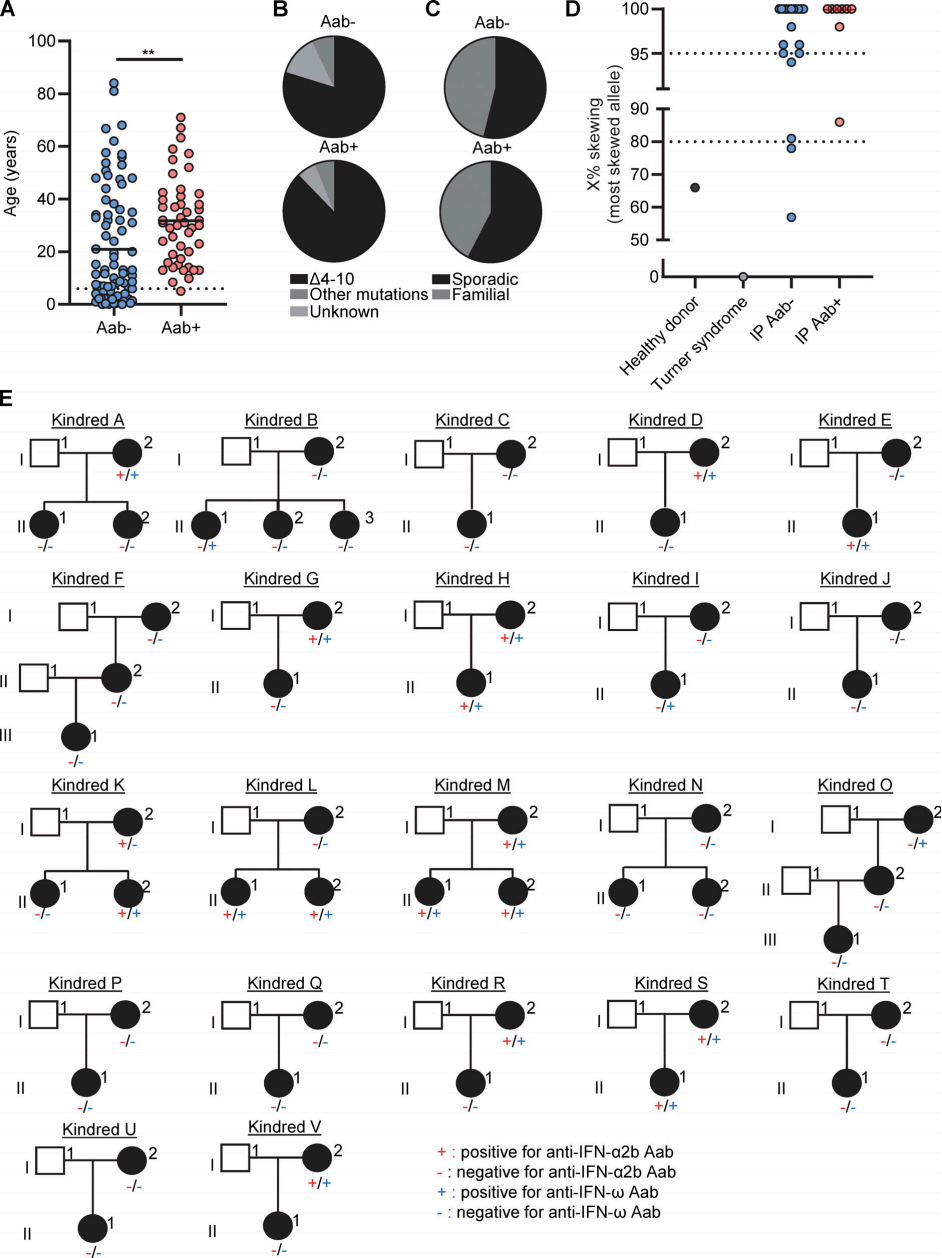
**Demographic, genetic, and familial features of female patients with IP by auto-Abs status. (A)** Age distribution of IP patients negative (Aab−) or positive (Aab+) for auto-Abs against type I IFNs. **(B–D)** Distribution of (B) underlying *NEMO* variants, (C) sporadic versus familial forms, and (D) X-chromosome inactivation skewing in female patients with IP negative (Aab−) or positive (Aab+) for auto-Abs against type I IFNs. **(E)** Auto-Ab status for IFN-α2a and IFN-ω in multiplex IP kindreds. Statistical analysis was performed with Student’s *t* tests. **P < 0.01.

We then investigated the possible correlation of positivity for auto-Abs against type I IFNs in IP patients with demographic, clinical, or genetic features. Autoimmunity is known to increase with age (Manoussakis et al., 1987), and the prevalence of auto-Abs against type I IFNs increases significantly after the age of 65 years (Bastard et al., 2021a). In this cohort of IP patients, (i) mean age was higher in patients positive for auto-Abs than in those without such antibodies (32 years versus 21 years, respectively) (Fig. 3 A). However, the prevalence of these antibodies remained stable from the age of 6 years onwards (∼40%) (Fig. 1 C). The distribution of *NEMO* genotypes was similar between patients positive and negative for auto-Abs (Fig. 3 B), and there was also no difference in the prevalence of auto-Abs between patients with sporadic and familial forms of IP (Fig. 3 C). X-chromosome inactivation was similarly skewed in both groups (Fig. 3 D). There was no correlation with clinical history, including the occurrence of the most severe manifestations of IP, such as CNS or ophthalmological involvement. Finally, in multiplex kindreds, auto-Abs could be found only in the oldest generation (kindreds A, D, G, R, and V), only in the youngest generation (kindreds B, E, I, and L), in both these generations (kindreds H, K, M, and S), or in neither of these generations (kindreds C, F, J, N, P, Q, T, and U) (Fig. 3 E). Overall, the prevalence of auto-Abs against type I IFNs remained stable, from the age of 6 years onwards, in IP patients, and was not correlated with age, underlying variant, mode of inheritance, kindred, or any clinical events related to IP disease.

### Small thymi with an abnormal structure in IP patients

**Figure S3.**
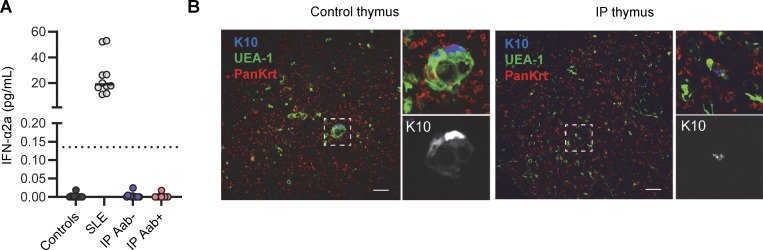
**Level of circulating IFN-α2a in females with incontinentia pigmenti (IP) and pathological study of the thymus from a male fetus with IP.** Fig. 4. **(A)** Determination of IFN-α2a levels with the Simoa platform for plasma from healthy donors (n = 10), patients with systemic lupus erythematosus (SLE, *n* = 10), and female patients with IP positive (*n* = 6) or negative (*n* = 12) for auto-Abs against type I IFNs. **(B)** Keratin 10 (K10), *Ulex europaeus* agglutinin 1 lectin (UEA-1), and pankeratin (PanKrt) staining of the thymus of a human male fetus with IP; comparison with an aged-matched control. Both thymi were obtained from fetuses at 19 wk of gestation with a similar estimated maceration time. Scale bar = 50 μm.

**Figure 4.**
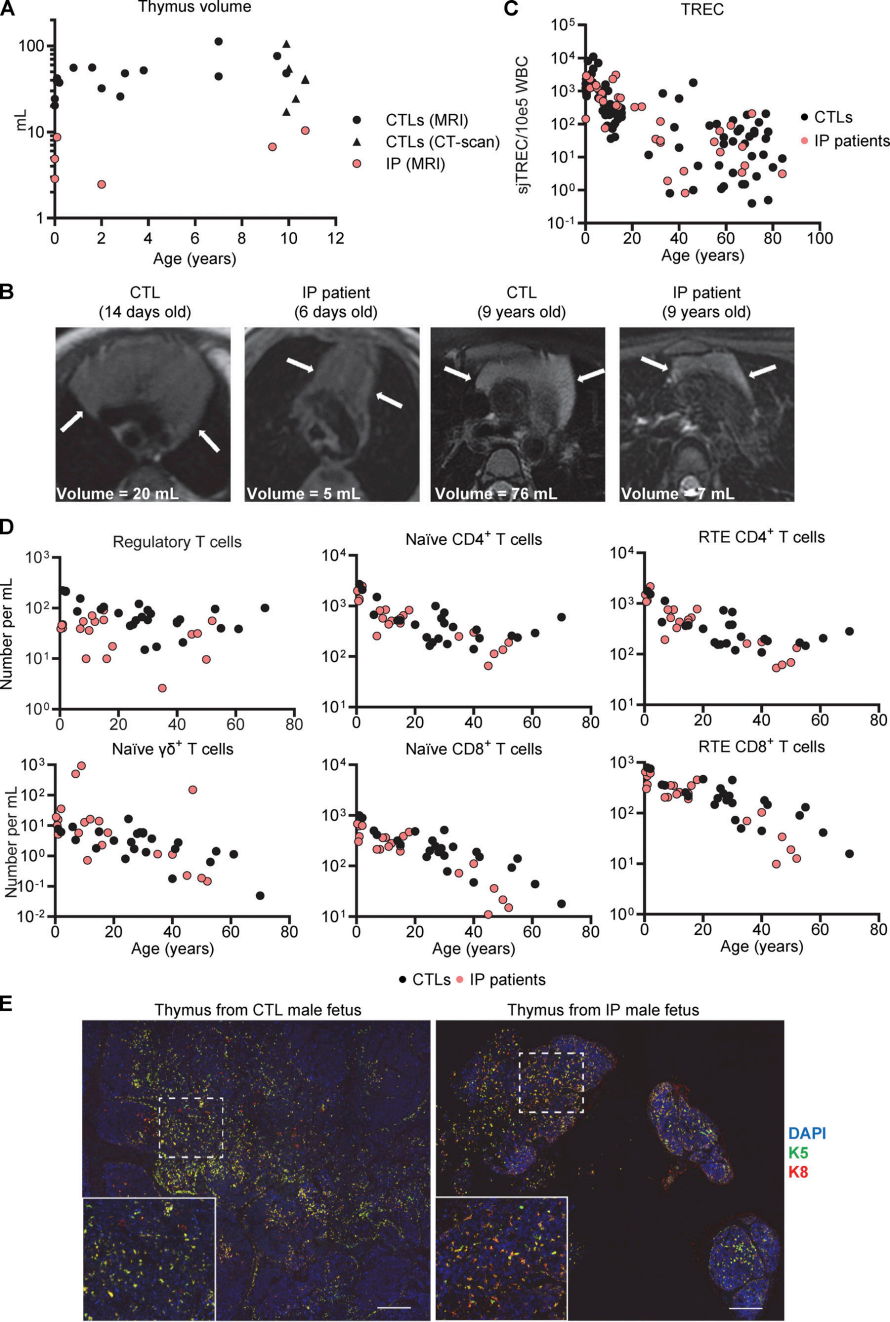
**Thymi of IP female patients and a male fetus. (A)** Thymus volume, assessed by MRI or computed tomography scan; *n* = 20 aged-matched controls and *n* = 6 IP patients. More details regarding controls and patients are provided in Table S6. **(B)** MRI images of thymi from IP patients and aged-matched controls of the indicated ages. **(C)** Quantification of TRECs in DNA extracted from the whole blood of healthy donors or patients with IP. Data were merged from three independent experiments. **(D)** Absolute counts of CD25^hi^CD127^low^ regulatory CD4^+^ T cells; naïve (CD45RA^+^CCR7^+^) γδ^+^, CD4^+^, or CD8^+^ T cells; and recent thymic emigrants (CD31^+^CD45RA^+^CCR7^+^) CD4^+^ or CD8^+^ T cells determined by mass cytometry on fresh whole blood from *n* = 24 female healthy donors and *n* = 23 IP patients. Experiments were performed independently and data were merged. **(E)** DAPI, keratin 5 (K5), and keratin 8 staining of formalin-fixed paraffin-embedded (FFPE) tissues from the thymus of a single male fetus with IP at 19 wk of gestation and of an aged-matched control. Scale bar = 200 μm.

We then investigated the mechanisms potentially underlying the production of these auto-Abs. We first tested the hypothesis that IP patients had a higher proportion of auto-Abs against type I IFNs due to higher levels of circulating IFN-α, as previously reported in systemic lupus erythematosus (Gupta et al., 2016; Howe and Leung, 2019; Panem et al., 1982; Rodero et al., 2017; Mathian et al., 2022). We assessed the levels of circulating IFN-α2 in plasma with a sensitive Simoa assay. We found that levels of circulating IFN-α2 were below the limit of detection in all IP patients, regardless of the presence or absence of auto-Abs (Fig. S3 A), as in healthy donors. Impaired thymic function underlies the presence of auto-Abs against type I IFNs in some IEI (Walter et al., 2015, 1; Bastard et al., 2021e; Schidlowski et al., 2022; Meager et al., 2006; Rosenberg et al., 2018; Maccari et al., 2017, 2; Ramakrishnan et al., 2018; Parsons et al., 2019; Le Voyer et al., 2023) and in patients with thymoma (Burbelo et al., 2010; Cheng et al., 2010). We therefore performed magnetic resonance imaging (MRI) to analyze the size and structure of the thymus in IP patients. Six IP patients under the age of 12 years had a smaller thymic volume than 21 age-matched controls (Fig. 4, A and B), with a mean of 10-fold decrease. In addition, all IP patients between the ages of 6 days and 10 years had thymi with straight margins, a feature generally found in adolescents (Manchanda et al., 2017). Conversely, all age-matched controls had thymi with convex margins (Fig. 4 B). All six IP patients in whom thymus size was assessed were tested for auto-Abs against type I IFNs and a positive result was obtained for one of these patients. However, thymus size was assessed before the age of 10 years, whereas these auto-Abs appear between the ages of 5 and 15 years (Fig. 1 C). We investigated the impact of this thymic hypoplasia on T cell development in female IP patients by quantifying a marker of thymic T cell output. T cell receptor excision circle (TREC) levels were within the control range (Fig. 4 C), as were the counts of recent thymic emigrant CD4^+^ or CD8^+^ T cells, naive CD4^+^ or CD8^+^ T cells, and naïve γδ T cells (Fig. 4 D). Counts of regulatory T cells were slightly low in IP patients. Thus, female IP patients had small thymi with a structure suggestive of premature senescence, but with normal levels of residual T cell production by the thymus.

### Small thymi with an altered structure in a human male fetus with IP and newborn female mice with IP

**Figure 5.**
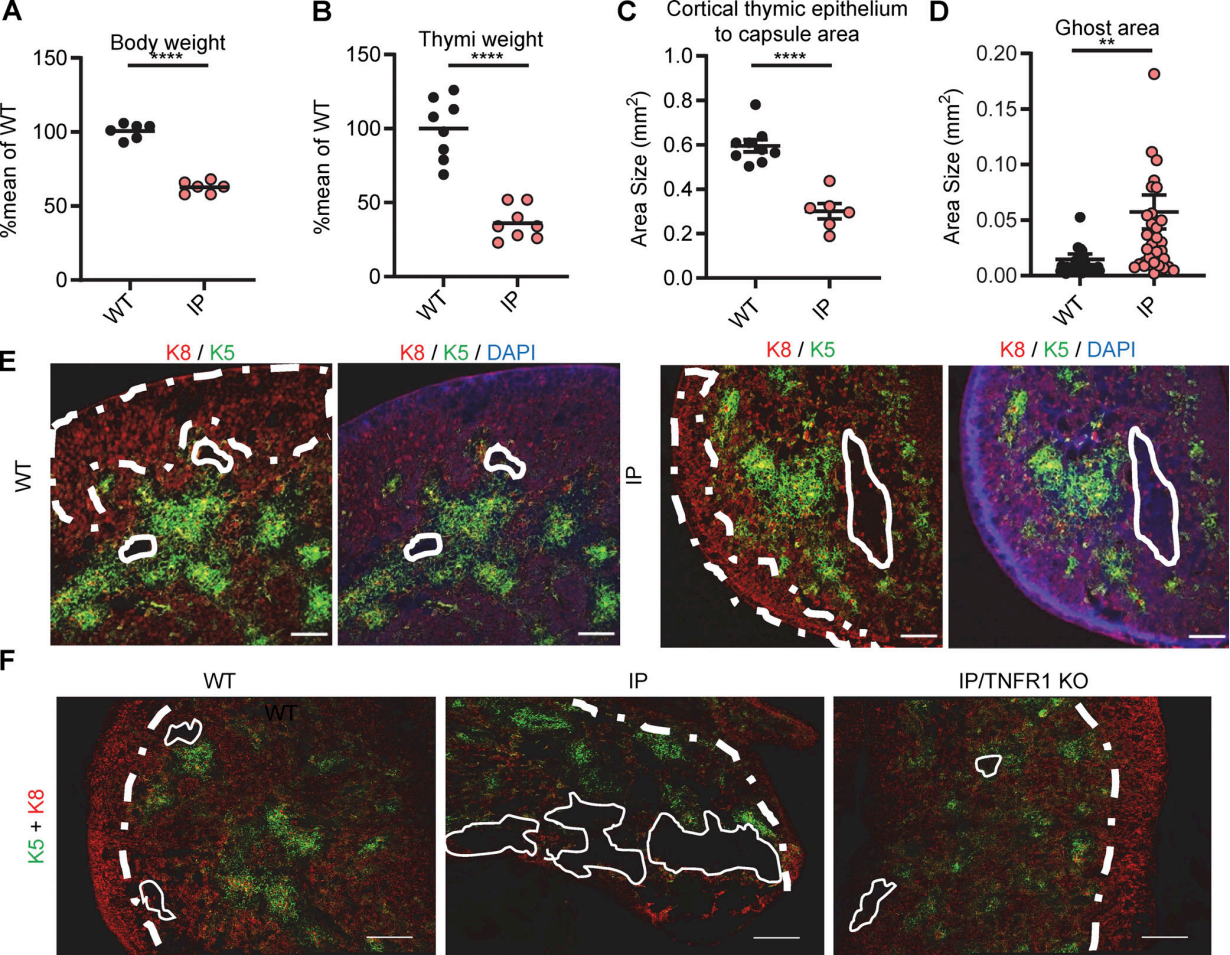
**Thymi of 7.5-day-old (P7.5) mice with IP. (A and B)** Weight of (A) the body and of (B) thymi from *n* = 9 WT mice and *n* = 9 IP mice. Results are expressed as a percentage of the mean for the WT in the same experiment. Data from two independent experiments are displayed. **(C)** Measurement of the area of the thymic cortex. **(D)** Measurement of the ghost area in thymi from WT and IP mice. **(E)** Representative confocal images of K8 (red), K5 (green), and DAPI (blue) staining of the thymus for WT and IP mice. Scale bar = 200 μm, *n* = 3–4 mice. Representative confocal images of K5/K8 (red), and CD4 (green) staining in WT and IP mice. Scale bar of left panel = 200 μm, Scale bar of other panel = 50 μm. *n* = 3–4 mice. **(F)** Representative images of K5/K8 (red) staining of thymi from WT mice (*n* = 4), *Nemo*^+/−^ female mice (IP, *n* = 3), and *Tnfr-1*^−/−^/*Nemo*^+/−^ female mice (IP/TNFR1 KO, *n* = 2). ns = not significant, P > 0.05; **P < 0.01; ****P < 0.0001. All experiments were performed at least two or three times independently.

**Figure S4.**
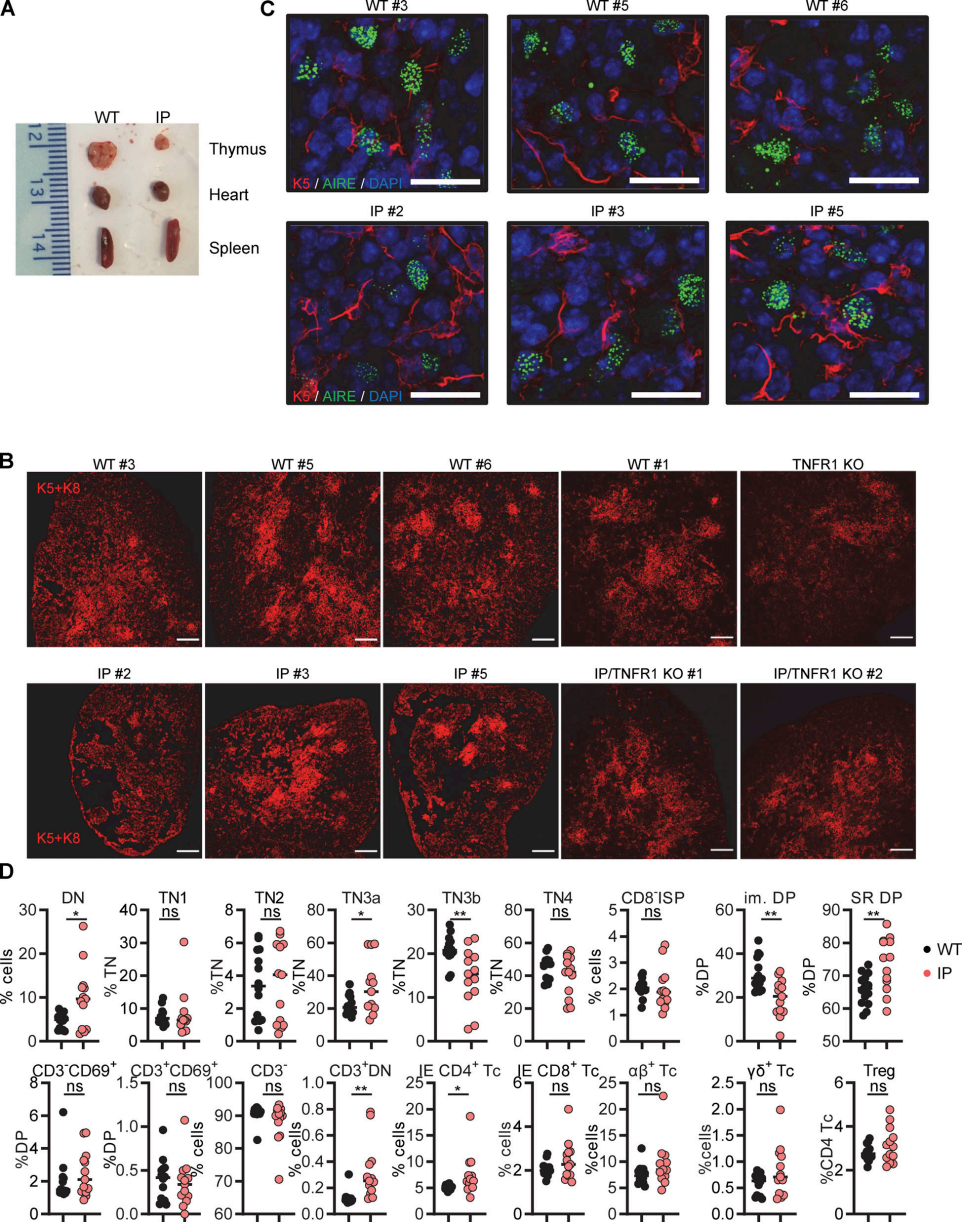
**Thymi of 7.5-day-old (P7.5) mice with IP.** Related to Fig. 5. **(A)** Images of the thymi, hearts, and spleens of a WT mouse and an IP mouse. **(B)** Representative images of K5/K8 (red) staining of thymi from WT mice (*n* = 4), *Nemo*^+/−^ female mice (IP, *n* = 3), *Tnfr-1*^−/−^/*Nemo*^+/+^ female mice (TNFR1 KO, *n* = 1), and *Tnfr-1*^−/−^/*Nemo*^+/−^ female mice (IP/TNFR1 KO, *n* = 2). Scale bar = 200 μm. **(C)** Representative confocal images of K5 (red), AIRE (green), and DAPI (blue) staining in WT and IP mice. Scale bar = 20 μm, *n* = 3–4 mice. **(D)** Percentages for the thymocyte subsets extracted from the thymi of *n* = 14 WT mice or *n* = 13 IP mice. DN = double-negative (CD4^−^CD8^−^); TN = triple-negative (CD3^−^CD4^−^CD8^−^), DP = double-positive (CD4^+^CD8^+^); ISP = immature single-positive; IE = immediate early. Statistical analysis was performed with Student’s *t* test. ns = not significant, P > 0.05; *P < 0.05; **P < 0.01. All experiments in mice were performed at least two or three times independently.

**Figure S5.**
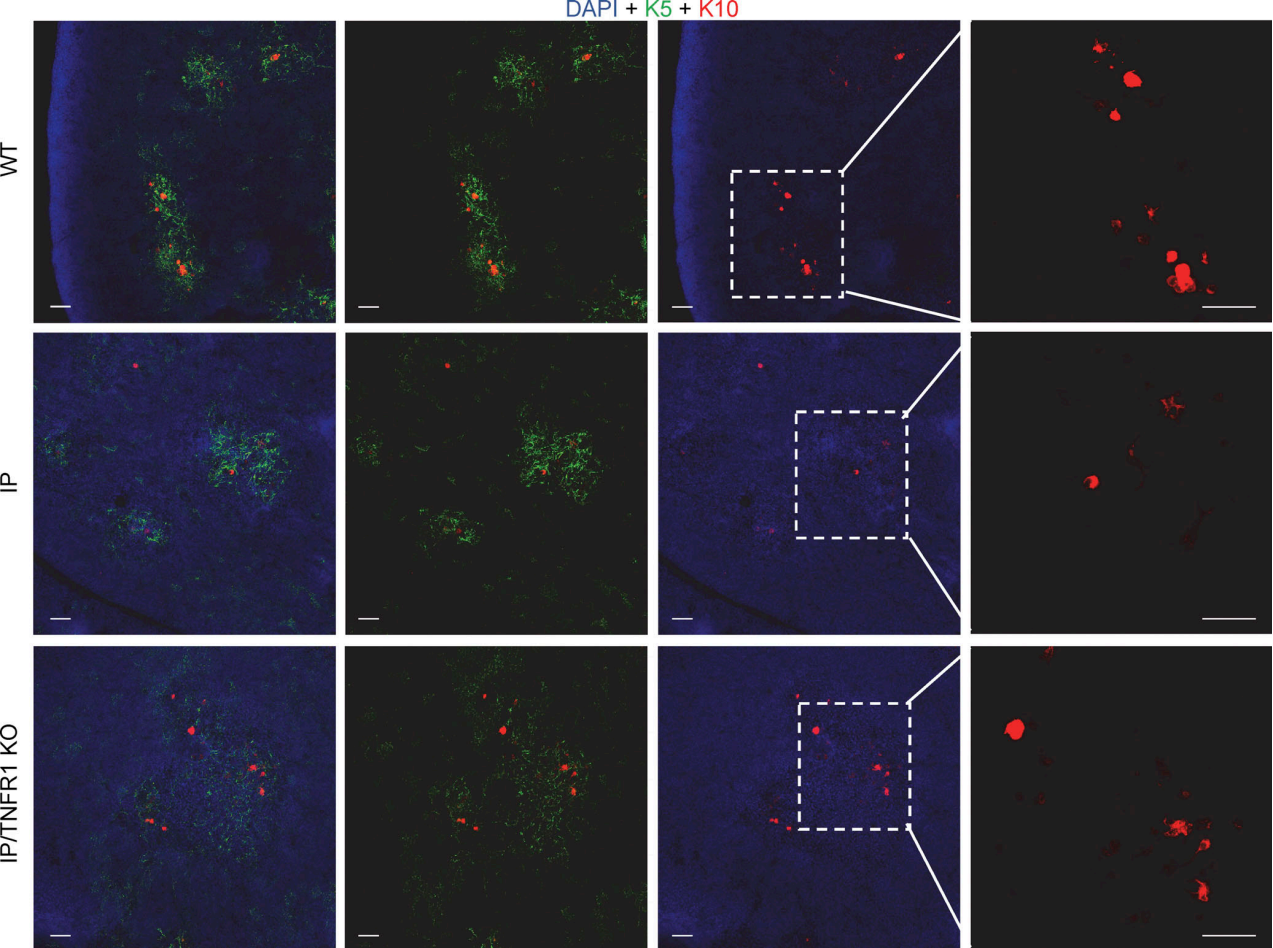
**Keratin 5 and 10 staining in thymi from newborn mice.** Related to Fig. 5. Keratin 5 (K5, green), keratin 10 (K10, red), and nuclear (DAPI, blue) staining of thymic sections from WT mice, IP mice, or IP/TNFR1 KO mice. Scale bar = 50 µm. Data representative from two independent experiments are displayed.

No thymic biopsy or necropsy specimens were available for the female IP patients. We analyzed the thymus of a 19-wk-old hemizygous male fetus with IP. We found significant hypoplasia, with a thymus weight of 0.01 g, one-fifth that of a normal thymus (0.05 g) (Martinovic, 2021) (Fig. 4 E). Conversely, other organs, such as the lungs, kidneys, heart, and testis, were of normal size and weight. Small numbers of mature keratin 10^+^ thymic epithelial cells (TECs) were also present (Fig. S3 B). We then performed histological studies of the thymus in a mouse model. The WT and mutant mice tested, including *Aire*^−/−^ mice, did not produce auto-Abs against type I IFNs, precluding studies of these auto-Abs in mice with IP (Pöntynen et al., 2006; Hubert et al., 2009; Kärner et al., 2013). Female mice with IP typically survive no more than 10 days, but they do have a clinical phenotype closely resembling that of IP patients (Makris et al., 2000; Schmidt-Supprian et al., 2000). We used 7-day-old female mice with IP for this study. As in IP patients, the thymus was smaller in mice with IP than in WT mice, whereas other organs, such as the spleen and heart, were of normal size (Fig. 5, A and B; and Fig. S4 A). We also found that the total mouse body weights and the corresponding thymus weights in female mice with IP were 63% and 40% decreased, respectively, compared to those in female WT mice (Fig. 5, A and B). The relative decrease in thymus weight was therefore greater than the relative decrease in body weight. We then performed immunofluorescence studies on the thymi of the mice. The staining of cross-sections for a cortical TEC (cTEC) marker, cytokeratin 8 (K8), showed that the thymic cortex area in mice with IP was one third that in WT control mice (Fig. 5, C and D). Thymus structure was also disorganized in IP mice, with three times as many ghost areas—areas depleted of TECs with no cortical or medullary cytokeratin staining—than in the control WT mice (Fig. 5, E and F; and Figs. S4 B and S5). These phenotypes of IP mice were rescued by TNFR1 receptor knockout (Fig. 5 F and Fig. S4 B), suggesting that the TECs underwent TNF-mediated cell death. AIRE expression was normal in the remaining medullary TECs (mTECs) (Fig. S4 C). We also performed flow cytometry on the thymic cells of WT and IP mice. Total cell counts were lower in IP mice than in the WT, consistent with the lower thymic weight (Fig. 5 B and Table S4). However, the distribution of thymocytes was normal overall, in terms of percentages, suggesting that the development of the remaining T cells was normal (Fig. S4 D). Thus, the thymi of IP mice displayed a TNF-mediated decrease in size and structural disorganization. Together with our findings for IP patients, these results suggest that IP leads to small dysplastic thymi in both mice and humans.

## Discussion

We report that at least 40% of female patients with IP carry auto-Abs neutralizing type I IFNs. This proportion was stable across a large international cohort, from the age of 6 years onward, suggesting that these auto-Abs appear in the first few years of life, subsequently remaining present throughout the individual’s life. In other words, their production is driven by IP itself, and not by age (Bastard et al., 2021e, 1). Our data suggest that the thymic hypotrophy and dysplasia observed in IP result from TNF-mediated cell death (Senegas et al., 2015; Pescatore et al., 2016; Pasparakis et al., 2002; Nenci et al., 2006), probably of TECs (Makris et al., 2000; Rudolph et al., 2000; Schmidt-Supprian et al., 2000). Conversely, TNF-deficient mice (Baseta and Stutman, 2000) and humans (Arias et al., 2024) have hypertrophic thymi with a normal structure. A follow-up study with a conditional knockout of NEMO in TECs may be helpful. Despite this limitation, the premature aging and thymic involution observed in IP probably underlie the development of auto-Abs against type I IFNs, given the strong association with age in the general population, the prevalence of these auto-Abs increasing sharply after 65 years of age (Bastard et al., 2021a). The clustering of IP patients into two groups of approximately equal size based on the presence or absence of auto-Abs against type I IFNs at a young age suggests that these auto-Abs may reflect the consequences of X-inactivation and variable mosaicism during thymic development (Parrish et al., 1996). Such a mechanism would explain why only about 40% of female IP patients produce auto-Abs against type I IFNs. It is also consistent with the heterogeneity observed for key clinical features of IP, such as ophthalmological and neurological phenotypes, which are also reported in about 30–50% of patients with IP (Hübner et al., 2022; Carney, 1976; Hadj-Rabia et al., 2003). Heterogeneity for these phenotypes is observed even within kindreds (Fusco et al., 2014; Hübner et al., 2022), like the presence of auto-Abs against type I IFNs in our cohort. Also consistent with this hypothesis, we observed similar differences in thymus size between IP mice and their WT littermates in a congenic mouse model. Our study therefore suggests that the thymus is a third pathogenic target of random X inactivation in IP female patients, together with the skin and the brain. IP patients are not considered to be prone to autoimmune disease (Fusco et al., 2014; Hübner et al., 2022; Bodemer et al., 2020). It is now tempting to speculate that their altered thymic architecture may contribute to the autoimmune or autoinflammatory manifestations anecdotally reported in some patients with IP (Yang et al., 2014; Piccoli et al., 2012; Poziomczyk et al., 2016; Endoh et al., 1996; Lin and Fu, 2010; Takada et al., 2010).

A pathogenic mechanism based on a thymic deficit underlying the production of auto-Abs against type I IFNs in female patients with IP is consistent with findings for patients with APS-1 (Bastard et al., 2021e; Schidlowski et al., 2022; Lemarquis et al., 2021; Meisel et al., 2021; Carpino et al., 2021; Beccuti et al., 2020; Ferré et al., 2021), inborn errors of the alternative NF-κB pathway (Maccari et al., 2017; Ramakrishnan et al., 2018; Parsons et al., 2019; Le Voyer et al., 2023), inborn errors of T cell development (such as hypomorphic RAG1 and RAG2 deficiencies [Walter et al., 2015], or IPEX [Rosenberg et al., 2018]), and thymoma (Burbelo et al., 2010; Cheng et al., 2010). IP and inborn errors of the alternative NF-κB pathway (Maccari et al., 2017; Ramakrishnan et al., 2018; Parsons et al., 2019; Le Voyer et al., 2023) are, however, somewhat unusual in the sense that (i) the auto-Abs produced are almost exclusively restricted to those neutralizing type I IFNs and (ii) T cell development is intrinsically normal. Histological analysis showed that mice with IP had a small thymic cortex, with structural abnormalities, including ghost areas, associated with a depletion of mTECs. This pattern resembles that observed in mice treated with an antibody antagonizing RANKL (Khan et al., 2014; Nakamura et al., 2022), an activator of both the classical and alternative NF-κB pathways. Conversely, the lack of auto-Abs against type I IFNs in patients with other inborn errors of the classical NF-κB pathway (Le Voyer et al., 2023) may reflect the impairment of antibody production in these patients (e.g.,, AR complete IKKβ deficiency [Burns et al., 2014; Mousallem et al., 2014] or AD NFKB1 deficiency [Li et al., 2021]), or the partial nature of the defect, which is insufficiently severe to increase sensitivity to TNF-mediated cell death (e.g.,, AD NFKB1 deficiency [Li et al., 2021], AD RelA deficiency [Comrie et al., 2018], or in male subjects with X-recessive partial NEMO deficiency [Döffinger et al., 2001]). Studies in humans suggest that the classical NF-κB pathway is essential for cTEC and mTEC survival, whereas the alternative NF-κB pathway is essential for mTEC development and maturation (Le Voyer et al., 2023).

Auto-Abs neutralizing IFN-α and/or IFN-ω have been linked to life-threatening COVID-19 pneumonia, influenza pneumonia, and an adverse reaction to YFV in IP patients. The eight IP female patients with IP and life-threatening viral diseases in our cohort carried auto-Abs neutralizing both IFN-α and/or IFN-ω. A causal link between these auto-Abs and these three viral infections has already been established in the general population (Bastard et al., 2020, 2021a, 2021d, 2021e; Zhang et al., 2022a; Hetemäki et al., 2021b; Busnadiego et al., 2022; Abers et al., 2021; Acosta-Ampudia et al., 2021; Chang et al., 2021; Chauvineau-Grenier et al., 2022; Goncalves et al., 2021; Koning et al., 2021; Solanich et al., 2021; Troya et al., 2021; Vazquez et al., 2021; van der Wijst et al., 2021; Schidlowski et al., 2022; Wang et al., 2021; Mathian et al., 2022; Lemarquis et al., 2021; Meisel et al., 2021; Savvateeva et al., 2021; Ziegler et al., 2021; Carapito et al., 2022; Credle et al., 2022; Eto et al., 2022; Frasca et al., 2022; Lamacchia et al., 2022; Raadsen et al., 2022; Simula et al., 2022; Soltani-Zangbar et al., 2022; Akbil et al., 2022; Borsani et al., 2022), and for COVID-19 in patients with APS-1 (Bastard et al., 2021e; Schidlowski et al., 2022; Lemarquis et al., 2021; Meisel et al., 2021; Carpino et al., 2021; Beccuti et al., 2020; Ferré et al., 2021) or with inborn errors of the alternative NF-κB pathway (Le Voyer et al., 2023). As in other conditions associated with the production of such auto-Abs, penetrance for life-threatening COVID-19 is high but incomplete (Bastard et al., 2021e; Schidlowski et al., 2022; Mathian et al., 2022; Lemarquis et al., 2021; Meisel et al., 2021; Carpino et al., 2021; Beccuti et al., 2020; Ferré et al., 2021; Le Voyer et al., 2023), with a much higher risk (10–1,000 times higher [Bastard et al., 2024a]) than that in the general population (Bastard et al., 2021a).

The penetrance of adverse reactions to live attenuated YFV also appears to be incomplete as only one of the five patients vaccinated developed the disease. This low penetrance may reflect the absence of IFN-β neutralization in addition to the neutralization of IFN-α and IFN-ω in IP patients, contrasting with two of the three previously reported patients with YFV adverse reactions (Bastard et al., 2021d). Alternatively, it may mean that they did not have auto-Abs against type I IFNs at the time of vaccination. The penetrance of severe influenza pneumonia was low in our cohort of auto-Ab-positive patients, as only one of the 44 patients was affected. These patients are probably prone to MERS (Raadsen et al., 2022) and to WNV encephalitis in endemic areas (Gervais et al., 2023). None of the patients had a history of adverse reaction to MMR, which has been reported in some patients with inherited IFNAR1 or IFNAR2 deficiency (Bastard et al., 2021c; Hambleton et al., 2013; Duncan et al., 2015). This may be due to a lack of auto-Abs when they were vaccinated at 12 mo of age, or because their auto-Abs do not neutralize IFN-β, or both. Patients with APS-1, whose auto-Abs against type I IFNs also appear in the first year of life and typically spare IFN-β, have not been reported to suffer from MMR disease either (Bastard et al., 2021e). In case of exposure to SARS-CoV-2, IP patients with auto-Abs that do not neutralize IFN-β may benefit from early treatment with IFN-β (Bastard et al., 2021b), monoclonal anti-SARS-CoV-2 antibodies (Lévy et al., 2021), antiviral drugs, or recombinant IFN-λ (Reis et al., 2023). Vaccination with SARS-CoV-2 RNA elicits a potent Ab response in patients with APS-1 (Sokal et al., 2023), suggesting that vaccination against COVID-19 is warranted in IP patients, together with annual influenza vaccination. To our knowledge, 10 auto-Abs-positive IP patients from our cohort were infected with SARS-CoV-2 after vaccination, and all of them developed asymptomatic or mild forms of infection. Patients with IP should be screened for anti-type I IFNs auto-Abs before the administration of live attenuated vaccines, such as the yellow fever vaccine. IP patients should be screened for these auto-Abs, which may also underlie other, as yet unknown viral diseases.

## Materials and methods

### Patients and healthy controls

Informed consent was obtained from patients in Brazil, Canada, Japan, United Europe, Serbia, and the United States, in accordance with local regulations and with institutional review board (IRB) approval. Experiments were conducted in France, Qatar, Sweden, and the United States of America in accordance with local regulations and with the approval of the IRB of the Rockefeller University and Inserm, for the United States of America and France, respectively. Healthy controls were recruited in France.

### Case reports for SARS-CoV-2 infections

P1 is a 32-year-old woman of Italian descent living in France previously reported to have suffered from critical COVID-19, but without publication of a detailed case report (patient 32 from Bastard et al. [2020]). In March 2020, P1 presented with a fever and cough. Bilateral opacities were observed on chest X-ray and PCR was positive for SARS-CoV-2. P1 was hospitalized for 5 days after symptom onset and was treated with 1–2 liters (L) O_2_/min, cefotaxime, spiramycin, acyclovir, and hydroxychloroquine. Computed tomography angiography (CTA) confirmed pneumonia (Fig. 2 A). An abrupt decline in the patient’s condition was observed 6 days later, necessitating support with 9 L O_2_/min. The patient was treated with tocilizumab. A progressive improvement was observed and the patient was discharged after 20 days of oxygen support. CTA performed 3 mo later showed an almost complete regression of pulmonary lesions, and plethysmography results were normal. P1 had auto-Abs neutralizing IFN-α and IFN-ω, both at 10 ng/ml, and the 13 IFN-α subtypes, but not IFN-β.

P2 is a 31-year-old-woman living in Canada for whom a case report has already been published by Rheault (Rheault, 2021). Briefly, the patient displayed severe COVID-19 pneumonia requiring oxygen support and she subsequently suffered from long-COVID.

P3 is 42 years old and is living in France. She was tested positive for SARS-CoV-2 by PCR after presenting with dyspnea in July 2020. She received nasal oxygen support without hospitalization. Auto-Abs against IFN-α and IFN-ω were detected, without auto-Abs against IFN-β, in a plasma sample collected 1 year before this episode. The patient’s plasma neutralized the 13 IFN-α subtypes.

P4 is a 38-year-old woman living in Italy. She tested positive for SARS-CoV-2 by PCR in March 2021. She was hospitalized due to dyspnea and received oxygen support (2 L/min for several days). 14 days after her positive PCR test for SARS-CoV-2, P4 was treated for 3 days with IFN-β Avonex (30 µg). She recovered and was discharged. P4 displayed auto-Abs neutralizing IFN-α and IFN-ω, both at 10 ng/ml, but not IFN-β. Her plasma completely neutralized all IFN-α subtypes except IFN-α4 and IFN-α6, which were partially neutralized.

P5 is a 38-year-old woman living in Italy. She presented interstitial pneumonia with positive PCR results for SARS-CoV-2. She did not require oxygen support. P5 tested positive for auto-Abs neutralizing IFN-α and IFN-ω, both at 10 ng/ml, but not for auto-Abs against IFN-β. Her plasma fully neutralized all IFN-α subtypes except IFN-α10 and IFN-α16, which were only partially neutralized.

P6 is a 29-year-old woman living in Italy. She presented pneumonia with a positive PCR test for SARS-CoV-2. She did not require oxygen support. P6 had auto-Abs neutralizing IFN-α and IFN-ω, both at 10 ng/ml, but not IFN-β. Her plasma fully neutralized all IFN-α subtypes except IFN-α5, IFN-α14, and IFN-α16, which were only partially neutralized.

P7 is a 52-year-old woman living in Italy. She presented mild COVID-19. She had auto-Abs against IFN-ω (100 pg/ml) only. Her plasma fully neutralized all IFN-α subtypes except IFN-α1, which was only partially neutralized.

P8 is a 30-year-old woman living in France. She presented mild COVID-19. Her plasma neutralized IFN-α and IFN-ω at 10 ng/ml, but not IFN-β.

P9 is a 24-year-old French nurse who was successfully pre-emptively treated with recombinant IFN-β after being diagnosed with COVID-19 (Bastard et al., 2021b). This patient has a high titer of auto-Abs against IFN-ω and a low titer of auto-Abs against IFN-α.

P10 is a 13-year-old girl living in Serbia. Serological results were positive for antibodies against antigens N and S but this patient presented no clinical event compatible with COVID-19. P10 had auto-Abs neutralizing 10 ng/ml of IFN-α and IFN-ω, but not IFN-β.

P11 is a 39-year-old woman living in France. In March 2020, at the age of 36 years, she presented dyspnea and tested positive for SARS-CoV-2. She was not hospitalized due to a lack of beds and did not receive oxygen support.

### Case reports for severe viral infections other than COVID-19

P12 is a 65-year-old woman living in Belgium with a history of hypothyroidism. She was vaccinated with live YFV at the age of 54 years. Following this vaccination, she presented progressive headache, asthenia, fever, and mild jaundice, leading to hospitalization 24 days after the injection. Blood test results were normal. Neurological examination revealed no abnormalities other than neck stiffness. The cerebrospinal fluid was clear and contained large numbers of cells, predominantly lymphocytes (21/mm^3^; 20% polymorphonuclear and 73% lymphocytes) and large amounts of protein (0.77 g/L) but normal glucose levels (0.65 g/L). The patient’s condition improved and she was discharged after 4 days of hospitalization. She remained convalescent for 2 mo. The condition presented by the patient met the criteria for yellow fever vaccine–associated neurological disease (YEL-AND) (Lecomte et al., 2020). Testing of a plasma sample collected 14 years after this episode revealed that this patient produced auto-Abs neutralizing IFN-α at 100 ng/ml and IFN-ω at 10 ng/ml.

P3 is a 42-year-old woman of Algerian descent living in France. She has a history of rheumatoid polyarthritis and presumed meningitis during infancy. At the age of 29 years, she was hospitalized 7 mo into pregnancy for left basal pneumonia caused by influenza virus A H_1_N_1_. She was successfully treated with oseltamivir, spiramycin, and cefotaxime and did not require ventilatory support. Tests on a plasma sample collected 12 years after this episode revealed that this patient produced auto-Abs neutralizing IFN-α at 100 ng/ml and IFN-ω at 10 ng/ml. P3 also had rheumatoid polyarthritis.

### Luciferase activity

The neutralization of IFN-α subtypes, IFN-β, and IFN-ω was assessed with a reporter luciferase assay, as previously described (Bastard et al., 2020, 2021a). Briefly, HEK293T cells were transfected with a pGL4.45 plasmid containing the firefly luciferase gene with five ISRE (5′-GGG​AAA​GTG​AAA​CTA-3′) in the promoter and a pRL-SV40 plasmid constitutively expressing the *Renilla* luciferase for normalization (#E2231; Promega). Cells were transfected in the presence of the X-tremeGene 9 transfection reagent (#6365779001; Roche) for 16 h. Cells in Dulbecco’s modified Eagle medium (DMEM; Thermo Fisher Scientific) supplemented with 2% fetal calf serum (FCS) and 10% healthy control or patient serum/plasma were either left unstimulated or were stimulated with IFN-α2 (#130-108-984; Miltenyi Biotec), IFN-ω (#SRP3061; Merck) at 10 ng/ml or 100 pg/ml, IFN-β (#130-107-888; Miltenyi Biotech) at 10 ng/ml, or IFN-α subtypes (Human IFN Alpha Sampler Set, #11002-1; PBL assay science) at 1 ng/ml for 16 h at 37°C. The Dual-Luciferase Reporter assay (#E1980; Promega) was performed according to the manufacturer’s protocol. Luminescence intensity was measured with a VICTOR X Multilabel Plate Reader (PerkinElmer Life Sciences). Firefly luciferase activity values were normalized against *Renilla* luciferase activity values. Values were then normalized against dual luciferase activity in the absence of stimulation. Samples were considered to be neutralizing if dual luciferase activity was below 3.

### IgG purification

IgG antibodies were purified on NAb Protein G Spin Columns (#89953; Thermo Fisher Scientific). Briefly, 100 μl plasma or serum was incubated with 400 μl Pierce Protein G IgG Binding Buffer (#21011; Thermo Fisher Scientific). Columns were washed four times with 400 μl phosphate-buffered saline (PBS), and IgG was eluted with 400 μl of 0.1 M glycine at pH 2.7. Eluted samples were immediately neutralized with 40 μl Tris 1.5 M pH 8. Purified IgG was concentrated on Pierce Protein Concentrators PES, 50K MWCO (#88504; Thermo Fisher Scientific). The protein concentration of the IgG-positive and -negative fractions was determined with a Nanodrop 2000 spectrophotometer (Thermo Fisher Scientific).

### X-chromosome inactivation

X-chromosome inactivation was assessed with a method derived from that of Allen et al. (1992) analyzing methylation of the *HUMARA* locus. Briefly, genomic DNA was extracted from whole blood and its concentration was determined with a Nanodrop 2000 spectrophotometer. Equal amounts (ranging from 300 to 1,000 ng) of DNA were digested overnight with 10 IU HpaII (specific digestion, #R0171L; New England BioLabs) or RsaI (control digestion, #R0167L; New England BioLabs) in a total volume of 25 μl CutSmart buffer (#B7204S; New England BioLabs) at 37°C. A second digestion with fresh RsaI or HpaII (10 IU in each case) was performed for 4 h at 37°C. Samples were then purified on multiwell plates (#8027; Pall Corporation) filled with Sephadex G-50 Superfine resin (#17-0041-01; GE Life Sciences). PCR was then performed with the GoTaq DNA polymerase (#M3005; Promega) and the following primers for *HUMARA*: forward 5′-TCC​AGA​ATC​TGT​TCC​AGA​GCG​TGC-3′ conjugated to FAM and reverse 5′-GCT​GTG​AAG​GTT​GCT​GTT​CCT​CAT-3′ unconjugated. The PCR products were then fixed by incubation with Hi-Di Formamide (#4311320; Applied Biosystems) in the presence of GenScan 500 ROX size standard (#401734; Thermo Fisher Scientific) at 95°C for 2 min. The fragments were then separated by capillary electrophoresis (#A30469; Applied Biosystems 3500xL; Thermo Fisher Scientific). Percent skewing was calculated as previously described (Thouin et al., 2003) by analyzing peak area with 3500 series data collection software 2 v2.0. DNA samples from a female patient with Turner syndrome, a male individual, and a female IP patient with known complete skewed XCI were used as positive controls for complete HpaI digestion. Digested DNA from patients with incomplete skewing was also run on a TBE-agarose gel in the presence of gel red to check for complete digestion. Ratios below 80:20 were considered to indicate random XCI, ratios of 80:20–90:10 were considered to indicate moderate skewing, and ratios >90:10 were considered to indicate extreme skewing (Jones, 2014).

### Protein microarrays

Proteome-wide auto-Ab screening was performed with microarrays (HuPro v4.0; CDI Laboratories) featuring full-length proteins expressed in yeast (*Saccharomyces cerevisiae*). Protein arrays were incubated for 90 min in 5 ml blocking buffer consisting of PBS with 2% bovine serum albumin and 0.05% Tween 20. The arrays were then incubated overnight in 5 ml of blocking buffer per array with serum from a blood donor or patient diluted 1:2,000. Each array was then washed five times for 5 min each with 5 ml PBST (PBS + 0.05% Tween 20). Alexa Fluor 647 goat anti-human IgG (Cat# A-21445, RRID:AB_2535862; Thermo Fisher Scientific) and Dylight 550 goat anti-GST (Cat# D9-1310; Columbia Biosciences Corporation) antibodies were diluted in blocking buffer (1:2,000 and 1:10,000, respectively), and each array was incubated with 5 ml of the resulting mixture for 90 min. Five washes were then conducted as previously described. Incubations and washes were performed on an orbital shaker with aluminum foil to block out light during the steps following the addition of fluorescent antibodies. Finally, each array was immersed in pure water three times and then centrifuged for ∼30 s for drying. The arrays were scanned later the same day on an Innoscan 1100AL fluorescence scanner (Innopsys) operated with Mapix software and the resulting images was analyzed with GenePix Pro 5.1.0.19. Data were normalized to compensate for signal intensity variation between experiments. Data for additional healthy controls from separate protein array experiments were included. Signal intensities were extracted from the scanned image with GenePix Pro 5.1.0.19 and the local background was subtracted:

$$Signal_{Protein}=\text{median}\;\left(Spot\;Pixel\;Intensity_{635}\right)-\left(Background\;Pixel\;Intensity_{635}\right)$$

Each protein was printed in duplicate spots. The resulting signal for a single sample is defined as:

$$Signal_{Sample}=\max\left\{ \begin{array}{l} Signal_{Protein}\;duplicate\;1\\ Signal_{Protein}\;duplicate\;2 \end{array}\right.$$

We eliminated spurious results by screening duplicates for large differences:

$${\text{Maximum}\;\text{duplicate}\;\text{discordance}}_{\text{protein}}=\max\left\{ \begin{array}{c} {Signal}_{Sample\;1}\;duplicate\;1-\;{Signal}_{Sample\;1}\;duplicate\;2\\ {Signal}_{Sample\;2}\;duplicate\;1-\;{Signal}_{Sample\;2}\;duplicate\;2\\ \ldots \\ {Signal}_{Sample\;n}\;duplicate\;1-\;{Signal}_{Sample\;n}\;duplicate\;2 \end{array}\right.$$

The mean signal intensity was calculated across case and control samples separately:

$$\mu_{SignalCases}=\sum_{i=1}^{n_{Cases}}Signal_{Sample}\;in\;cases$$

Proteins with discordant duplicates (i.e., Log_2_FC > 5 between duplicates), which were more prone to artifacts, were excluded.

### Bead-based protein array

Bead-based protein assays were performed as previously described (Le Voyer et al., 2023) with the relevant antigens displayed in Table S5.

### PhIP-seq

We calculated species-specific significance cutoff values to estimate the minimum number of enriched, non-homologous peptides required to consider a sample seropositive (as previously described [Xu et al., 2015]) with an in-house dataset and a generalized linear model. For each sample, we calculated virus-specific scores by dividing the counts of enriched, non-homologous peptides by the estimated cutoff score. These adjusted virus scores were used in the heatmap plot. We calculated and plotted the mean antibody responses of both patients and a previously described pediatric control cohort of lean individuals without infectious or immunological disease (*n* = 111; age range: 7–15 years; median age: 11.0 years) (Hasan et al., 2021; Khan et al., 2021). Pooled human plasma used for IVIg (Privigen CSL Behring AG) and human IgG-depleted serum (Molecular Innovations, Inc.) were used as additional controls. All research on human subjects was performed after informed written consent had been obtained or on de-identified samples. The procedures were approved by the institutional research ethics board of Sidra Medicine.

### Determination of plasma IFN-α concentration with the Simoa platform

Plasma IFN-α levels were determined as previously described (Bastard et al., 2020) with the Simoa HD1 Analyzer for patients and healthy donors, in accordance with the manufacturer’s instructions (Quanterix).

### Screening for auto-Abs against native DNA, extractable DNA antigens, and tissue antigens

Screening for auto-Abs against native DNA, extractable DNA antigens, and tissue antigens was performed as previously described (Bastard et al., 2021d). Screening for auto-Abs against native DNA, extractable DNA antigens, and tissue antigens was performed as previously described (Bastard et al., 2021d). Briefly, anti-native DNA detection was performed by indirect immunofluorescence methods on the flagellate organism *Crithidia luciliae* with the IgG Theradiag kit (#ME 0296) and ELISA for Ab quantification with the anti–double-stranded DNA IgG kit on ETI-MAX 3000 Equipment from DRG International (#EIA-3566). Extractable nuclear antigen Abs were detected by ELISA (RNP, Sm, SSA/Ro, Trim 21 [SSA/Ro 52 kDa], SSB/La, Scl70, centromere B and Jo-1) with the ANAScreen Kit (#ORG 238; Orgentec) or the immunodot EUROLINE ANA Profile 3 Plus DFS70 (IgG) kit (#1590-30; Euroimmun). auto-Abs against smooth muscles, mitochondria, and LKM1 were detected by indirect immunofluorescence methods on triple rodent tissues with a kit from Theradiag (#ME 0832) and/or by immunodot methods with the EUROLINE ANA Profile 3 Plus DFS70 (IgG) kit (#1590-30; Euroimmun) for mitochondria type 2.

### TRECs

Signal joint TRECs (sjTRECs) were quantified by nested multiplex qPCR with a method adapted from that of Dion et al. (2007). Briefly, multiplex PCR amplification of both the sjTREC and the CD3γ chain, used as a housekeeping gene, was performed on DNA extracted from whole blood in a final volume of 100 μl. The cycling conditions were as follows: 10 min of initial denaturation at 95°C, then 22 cycles of 30 s at 95°C, 30 s at 60°C, and 2 min at 72°C. The outer 3′/5′ primer pairs were used as previously described (Dion et al., 2007). The qPCR conditions in LightCycler experiments, using the inner primer pairs previously described (Dion et al., 2007) and performed on 1/100th of the initial PCR products, were as follows: 1 min of initial denaturation at 95°C, then 40 cycles of 1 s at 95°C, 10 s at 60°C, and 15 s at 72°C. Fluorescence signals were assessed at the end of the elongation steps. sjTREC and CD3γ LightCycler quantifications were performed in independent experiments with the same first-round serial dilution standard curve.

### Thymus imaging

To assess the volume of the thymus, we used methods described elsewhere (Le Voyer et al., 2023; Materna et al., 2024). Briefly, the thymus was measured in three planes: (i) thickness, (ii) width in the axial plane through the aortic arch, and (iii) greatest height in a coronal or sagittal oblique plane. Thymic volume was estimated with the following formula: thickness × width × height. Detailed information about the patients and the age-matched controls is provided in Table S6.

### Mass cytometry of whole blood

Mass cytometry of fresh whole-blood cells was performed as previously described with a homemade customized panel (Materna et al., 2024). All samples were processed within 36 h of blood sampling.

### Male fetal thymus examination

Having obtained parental consent, we used a standard fetopathology protocol (Martinovic, 2021) to investigate a male fetus stillborn at 19.2 wk of gestation. The fetus was hemizygous for the recurrent *NEMO* deletion. Based on gross and histological criteria, maceration was estimated at about 3 days. Two male fetuses with a similar age of development and level of maceration were used as controls. Tissues were fixed in formalin and embedded in paraffin. Staining was performed as follows. Antigen retrieval was performed on rehydrated tissue by boiling sections in Citra antigen retrieval solution (Biogenex). The sections were blocked by incubation for 30 min at room temperature in a blocking solution (CAS-Block [Thermo Fisher Scientific] + 0.2% Triton X-100 [Sigma-Aldrich] +1%BSA +5% donkey serum). Sections were then incubated overnight at 4°C with primary antibodies. The following antibodies were used: KRT5 Alx488 Rb (clone EP1601Y, #ab193894; Abcam), KRT8-Alx647 b (clone EP1628Y, #ab192468; Abcam) pan cytokeratin (clones AE1/AE3 + 5D3, #CM162A; Biocare medical), KRT10-Alx647 (clone EP1607IHCY, #ab194231; Abcam), and UEA-1 bio (#B-1065-2; Vector Laboratories). The sections were washed with 0.1% Tween in PBS and stained by incubation with secondary antibodies for 1 h at room temperature. The sections were washed with 0.1% Tween in PBS and mounted in ProLong Diamond Antifade mounting solution (Thermo Fisher Scientific). Images were acquired on a Leica Thunder microscope.

### Mice

*Nemo*^wt/ko^ and wt female mice were produced by crossing *Nemo*^wt/fl^ females with Cre-Deleter (X^cre^/Y) males as previously described (Schmidt-Supprian et al., 2000). The pups were killed at day 7 (P7) and their thymi were collected and snap-frozen in an optimal cutting temperature (OCT) medium for freezing and storage at −70°C until analysis. Genotyping was performed as previously described (Schmidt-Supprian et al., 2000). *Nemo*^wt/ko^/*Tnfr1*^ko/ko^ female pups were produced by crossing *Nemo*^wt/fl^/*Tnfr1*^wt/ko^ females with Cre-Deleter/*Tnfr1*^ko/ko^ males (*Tnfr1*^ko/ko^ Strain #002818; Jackson Labs). All experiments involving mice were conducted in accordance with the regulations of CEA Grenoble and in compliance with French legislation and the European Union Directive of 22 September 2010 (2010/63/UE). Mice were reared under specific pathogen–free conditions in the animal facilities of CEA Grenoble.

### Flow cytometry on murine thymi

Multiparametric immunophenotyping was performed at the CIPHE-PHENOMIN (US012; Inserm) flow cytometry facility. Thymi were collected from 6-to 7-day-old animals with extraction according to the IMPRESS protocol (https://www.mousephenotype.org/impress/protocol/174/7). Briefly, organs were disrupted on an OctoGentleMACS system (Miltenyi Biotec) with 600 Mendel units of collagenase D (Roche Life Science) and 30 μg DNAse I (Sigma-Aldrich) for 20 min at room temperature. The resulting cell suspension was filtered and the cells in the crude extract were counted on an Attune NxT (Thermo Fisher Scientific) volumetric cytometer. Before staining, cells were incubated for 10 min on ice with the anti-CD16/32 (2.4G2) antibody to block Fc receptors. In all experiments, DAPI (Invitrogen) was used to exclude dead cells from the analysis. Multiparameter FACS acquisition was performed on a Fortessa LSRII SORP machine (BD Biosciences). The analysis was performed with FACSDiva 9.01 (BD Biosciences) software and Cytobank 10.1 (Beckman Coulter). Doublets were systematically excluded on the basis of side scatter (SSC) and forward scatter (FSC). The antibodies used for immunophenotyping are listed in Table S7.

### Immunofluorescence staining and imaging

Snap-frozen mouse thymic tissues were embedded in OCT Compound (4583; Tissue-Tek) and stored at −80°C. Sections (50 µm) were then cut on a cryostat (Leica) and dried on Superfrost Plus (1255015; Thermo Fisher Scientific) slides before fixation by incubation with 4% paraformaldehyde (28908; Thermo Fisher Scientific) in PBS for 15 min at room temperature. The slides were then transferred to Immunomix solution containing 0.3% Triton X-100 (Sigma-Aldrich), 0.2% BSA (Sigma-Aldrich), and 0.1% sodium azide (Sigma-Aldrich) in PBS. Slides were then rehydrated by incubation in PBS for 5 min before permeabilization by incubation in Immunomix solution for 1 h, with shaking, at room temperature. The sections were then blocked with BlockAid (B10710; Thermo Fisher Scientific) and stained with primary antibody for 3 h at room temperature. The slides were then stained with DAPI for 5 min at room temperature and then washed four times in 1× PBS for 5 min each.

All sections were mounted in ProLong Diamond Antifade Mountant (Thermo Fisher Scientific). Images were acquired on a Leica SP5 (Leica) laser scanning confocal microscope. Thymic cortical-to-capsule and ghost area images were analyzed and measurements were made with Imaris software. The following antibodies were used in this study: KRT5-Alexafluor488 (EP1601Y, Cat#193894; Abcam), KRT5-Alexafluor647 (EP1601Y, Cat#193895; Abcam), KRT8 (EP1628Y, Cat# 192468; Abcam), CD4-Alexafluor488 (RM4-5, Cat# 100529; Biolegend), and Aire-Alexafluor488 (5H12, Cat# 53593482; Thermo Fisher Scientific) and DAPI (422801; Biolegend).

### Statistical analysis

Statistical significance was assessed in an unpaired, parametric, two-tailed Student’s *t* test.

### Online supplemental material

Fig. S1 shows additional data regarding auto-Abs in female patients with incontinentia piemgnti. Fig. S2 shows PhIP-Seq viral antibody responses. Fig. S3 shows IFN-α2a levels in plasmas of patients or controls, and immunofluorescence staining of a thymus of a human male fetus with an aged-matched control. Fig. S4 and Fig. S5 show additional macroscopic, flow cytometry, and immunofluorescence data in murine model. Table S1 shows the summary of the characteristics of the patients from the cohort. Table S2 shows data for microarray testing of auto-Ab reactions in healthy donors and IP patients. Table S3 shows data from a bead-based protein array for the indicated cytokines in healthy donors and IP patients. Table S4 shows flow cytometry data for thymocytes extracted from WT and IP thymi. Table S5 shows references to proteins used for bead-based protein assays. Table S6 provides details of patients and age-matched controls for thymus imaging. Table S7 shows antibodies used for murine flow cytometry.

## Supplementary Material

Table S1 shows a summary of the characteristics of the patients from the cohort.

Table S2 shows data for microarray testing of auto-Ab reactions to 20,052 native monomeric proteins in 20 healthy donors and 25 IP patients.

Table S3 shows data from a bead-based protein array for the indicated cytokines in *n* = 93 HD, and *n* = 30 and *n* = 24 IP patients negative and positive, respectively, for auto-Abs against type I IFNs in neutralization assays.

Table S4 shows flow cytometry data for thymocytes extracted from WT and IP thymi.

Table S5 shows proteins used for bead-based protein assays.

Table S6 shows details of patients and age-matched controls for thymus imaging.

Table S7 shows antibodies used for murine flow cytometry.

## Data Availability

The data are available from the corresponding authors upon reasonable request. All the data needed to evaluate the conclusions of the paper are present in the paper or the online supplemental material.
