# Supplementary material for: Incontinentia pigmenti underlies thymic dysplasia, autoantibodies to type I IFNs, and viral diseases
Source: J Exp Med. 2024 Oct 1;221(11):e20231152. doi: 10.1084/jem.20231152 (PMC11448874; doi:10.1084/jem.20231152)
Supplement: Table S1 — shows a summary of the characteristics of the patients from the cohort. [file JEM_20231152_TableS1.docx]

**Table S1 – Summary of the characteristics of the patients from the cohort**

| **Characteristics** | **Percentage (number of patients/number of patients for whom information was available)** |
| --- | --- |
| ***Sex*** |  |
| *Female* | 100% (131/131) |
| *Male* | 0% (0/131) |
| ***Country of residence*** |  |
| *Belgium* | 5% (7/131) |
| *Brazil* | 5% (7/131) |
| *Canada* | <1% (1/131) |
| *France* | 50% (65/131) |
| *Germany* | 2% (2/131) |
| *Italy* | 17% (22/131) |
| *Japan* | 5% (7/131) |
| *Serbia* | 10% (13/131) |
| *Switzerland* | <1% (1/131) |
| *United States of America* | 5% (6/131) |
| ***Clinical manifestations of IP*** |  |
| *Cutaneous* | 100% (131/131) |
| *Ophthalmological* | 34% (35/103)) |
| *Neurological* | 30% (32/105) |
| ***Inheritance*** |  |
| *Sporadic* | 55% (47/85) |
| *Familial* | 45% (38/85) |
| **IKBKG/NEMO *variants*** |  |
| *Δ4-10* | 82% (108/131) |
| *Other variants* | 11% (14/131) |
| *Unknown* | 7% (9/131) |
