# Supplementary material for: Incontinentia pigmenti underlies thymic dysplasia, autoantibodies to type I IFNs, and viral diseases
Source: J Exp Med. 2024 Oct 1;221(11):e20231152. doi: 10.1084/jem.20231152 (PMC11448874; doi:10.1084/jem.20231152)
Supplement: Table S5 — shows proteins used for bead-based protein assays. [file JEM_20231152_TableS5.docx]

**Table S5 – Proteins used for bead-based protein assays**

| **Protein** | **Company** | **Cat.** |
| --- | --- | --- |
| IFNA1 | Sigma | SRP4596 |
| IFNA2 | Origene | TP321091 |
| IFNA4 | Origene | TP323649 |
| IFNA5 | Origene | TP310825 |
| IFNA6 | Origene | TP760329 |
| IFNA7 | Novus | 11079-IF |
| IFNA8 | Origene | TP311169 |
| IFNA10 | Origene | TP314055 |
| IFNA14 | Prospec | cyt-135-b |
| IFNA16 | Novus | 11190-1 Lot# 6865 |
| IFNA17 | Origene | TP320824 |
| IFNA21 | Origene | TP310115 |
| IFNB1 | MedChemExpress | HY-P73128 |
| IFNW1 | Origene | TP721113 |
| IFNE | R&D | 9667-ME/CF |
| IFNK | Cusabio | CSB-EP889172HU |
| IFNG | MedChemExpress | HY-P7025 |
| IL28a | Nordic biosite (Sino Biological) | 12340-H0By |
| IL28b | Abcam | ab276441 |
| IL29 | Abcam | ab155625 |
| IFNL4 | R&D | 9165-IF |
| IL12 | Miltenyi Biotec | 130-129-718 |
| IL23 (IL12+p19) | MedChemExpress | HY-P73193 |
| IL17A | Origene | TP318057 |
| IL17F | Origene | TP723203 |
| IL22 | Miltenyi Biotec | 130-096-297 |
| IL6 | MedChemExpress | HY-P7044G |
| TNF alpha | MedChemExpress | HY-P7416 |
