# Supplementary material for: Incontinentia pigmenti underlies thymic dysplasia, autoantibodies to type I IFNs, and viral diseases
Source: J Exp Med. 2024 Oct 1;221(11):e20231152. doi: 10.1084/jem.20231152 (PMC11448874; doi:10.1084/jem.20231152)
Supplement: Table S6 — shows details of patients and age-matched controls for thymus imaging. [file JEM_20231152_TableS6.docx]

**Table S6 – Details of the patients and age-matched controls used for thymus imaging**

| **Status** | **Sex** | **Age** | **MRI or CT** | **Reasons for imaging** |
| --- | --- | --- | --- | --- |
| IP1 | F | 6 d | MRI | Brain seizure |
| IP2 | F | 19 d | MRI | Investigation after a fall |
| IP3 | F | 6 d | MRI | IP investigation, skin disease |
| IP4 | F | 2 y | MRI | IP investigation, skin disease |
| IP5 | F | 9.3 y | MRI | This study (prospective) |
| IP6 | F | 10.7 y | MRI | This study (prospective) |
| CTL1 | M | 10 d | MRI | Esophageal duplication |
| CTL2 | F | 14 d | MRI | Cystic hygroma |
| CTL3 | M | 30 d | MRI | Sternal tumor |
| CTL4 | M | 1 m | MRI | Cystic lymphangioma |
| CTL5 | M | 2 m | MRI | Lung lesions |
| CTL6 | F | 10 m | MRI | Lung atresia |
| CTL7 | F | 19 m | MRI | Asymptomatic sequestration |
| CTL8 | M | 2 y | MRI | Cystic hygroma |
| CTL9 | M | 2.8 y | MRI | Diaphragmatic sequestration |
| CTL10 | M | 3 y | MRI | Trauma |
| CTL11 | M | 3.8 y | MRI | Neuroblastoma |
| CTL12 | M | 7 y | MRI | Long-term follow-up of echinococcosis |
| CTL13 | F | 7 y | MRI | Cervicothoracic cystic lymphangiomas |
| CTL14 | F | 9.5 y | MRI | Asymptomatic cystic fibrosis |
| CTL15 | F | 9.9 y | MRI | Cystic lymphangiomas |
| CTL16 | F | 9.9 y | CT | Polytrauma |
| CTL17 | F | 9.9 y | CT | Polytrauma |
| CTL18 | F | 10.0 y | CT | Polytrauma |
| CTL19 | F | 10.3 y | CT | Polytrauma |
| CTL20 | F | 10.7 y | CT | Polytrauma |
| CTL21 | F | 10.7 y | CT | Polytrauma |

IP = incontinentia pigmenti; CTL= control; d = days; m = months; y = years; MRI = magnetic resonance imaging; CT-scan = computed tomography scan. The results for the controls are consistent with the variability of thymus volume reported in previous studies (Steinmann, 1986; Francis et al., 1985; Yekeler et al., 2004). The IP patients were tested for type I IFN-neutralizing activity at the ages of 1, 6, 8, 9 or 10 years. The patient tested at the age of 8 years (IP5 below) tested positive; all the others tested negative.
