# Supplementary material for: Incontinentia pigmenti underlies thymic dysplasia, autoantibodies to type I IFNs, and viral diseases
Source: J Exp Med. 2024 Oct 1;221(11):e20231152. doi: 10.1084/jem.20231152 (PMC11448874; doi:10.1084/jem.20231152)
Supplement: Table S7 — shows antibodies used for murine flow cytometry. [file JEM_20231152_TableS7.docx]

**Table S7 – Antibodies used for murine flow cytometry**

| **Specificity** | **Clone** | **Company** |
| --- | --- | --- |
| CD4 | RM4.5 | BD Biosciences |
| CD5 | 53-7.3 | BD Biosciences |
| CD44 | IM7 | Biolegend |
| CD69 | H1.2F3 | BD Biosciences |
| CD8a | 53-6.7 | BD Biosciences |
| Qa2 | 695H1.9.9 | Biolegend |
| CD117 | 2B8 | BD Biosciences |
| TCRd | GL3 | e-Bioscience |
| CD3e | 145-2C11 | BD Biosciences |
| CD71 | R17217 | Biolegend |
| CD335 | 29A1.4 | BD Biosciences |
| CD24 | M1.69 | BD Biosciences |
| CD25 | PC61 | Biolegend |
|  |  |  |
